# A maize phytochrome‐interacting factors protein ZmPIF1 enhances drought tolerance by inducing stomatal closure and improves grain yield in *Oryza sativa*

**DOI:** 10.1111/pbi.12878

**Published:** 2018-03-12

**Authors:** Yong Gao, Meiqin Wu, Mengjiao Zhang, Wei Jiang, Xiaoyun Ren, Enxing Liang, Dongping Zhang, Changquan Zhang, Ning Xiao, Yan Li, Yi Dai, Jianmin Chen

**Affiliations:** ^1^ Jiangsu Key Laboratories of Crop Genetics and Physiology and Plant Functional Genomics of the Ministry of Education Yangzhou University Yangzhou Jiangsu China; ^2^ Lixiahe Region Agricultural Scientific Research Institute of Jiangsu Yangzhou Jiangsu China; ^3^ State Key Laboratory of Crop Biology College of Agronomy Shandong Agricultural University Taian China

**Keywords:** drought tolerance, water saving, morphological character, physiological trait, transcription factor, stomata

## Abstract

Phytochrome‐interacting factors (PIFs) play major roles in regulating plant growth and development, but their roles in drought stress remain elusive. Here, we cloned and characterized a maize (*Zea mays*) PIF transcription factor, *ZmPIF1*. The expression level of *ZmPIF1* was significantly induced by independent drought and abscisic acid (ABA) treatments. The *ZmPIF1* transgenic rice and *Arabidopsis* displayed water saving and drought resistance, which were associated with reduced a stomatal aperture and transpiration rate. Moreover, the *ZmPIF1* transgenic rice were hypersensitive to exogenous ABA, while the endogenous ABA level was not significantly changed, suggesting that *ZmPIF1* was a positive regulator of the ABA signalling pathway. Digital gene expression (DGE) results further indicated that *ZmPIF1* participated in ABA signalling pathway and regulated the stomatal aperture in rice. In addition, grain yield and agronomic traits analysis over 4 years showed that *ZmPIF1* was able to increase the grain yield through an increase in tiller and panicle numbers in transgenic rice. Overall, *ZmPIF1* plays an important role in the ABA‐mediated regulation of stomatal closure to control water loss. *ZmPIF1* can enhance water saving and drought resistance and improve the crop yield in rice, illustrating the capacity of *ZmPIF1* for crop improvement.

## Introduction

Global warming is expected to increase the frequency and intensity of droughts worldwide. Additionally, water scarcity is a major agricultural problem, restricting crop expansion and reducing crop yield. Of the total human water consumption, 70% is agricultural water. Thus, adaptation and mitigation strategies should focus on researching the mechanism of crop water saving and drought resistance to reduce current and future drought risks. In fact, even a small improvement in crop water saving will result in large reductions in crop loss (Claeys and Inze, 2013).

Stress avoidance and stress tolerance are two different mechanisms for dealing with low water availability, and both responses are related to abscisic acid (ABA)‐dependent and ABA‐independent mechanisms (Claeys and Inze, 2013; Lawlor, 2013). In plants, ABA is a main regulator of the transpiration rate and other mechanisms of water saving and drought resistance (Jones, 2015). It has been extensively shown that plants accumulate more ABA, which controls stomatal closure under drought conditions, thereby saving water (Murata *et al*., 2015).

Phytochrome‐interacting factors (PIFs) are a subset of basic helix‐loop‐helix (bHLH) transcription factors. The first *PIF* gene cloned from bHLH transcription factors was *PIF3* (Ni *et al*., 1998). Subsequently, *PIF1*,* PIF4*,* PIF5*,* PIF6*,* PIF7* and *PIF8* were identified (Leivar and Monte, 2014; Leivar and Quail, 2011). All of these PIFs contain a conserved bHLH domain that is responsible for DNA binding. These PIFs also contain an active phytochrome B (APB) motif, which is conserved in the N‐terminal sequence and necessary for phytochrome B (phyB)‐specific binding. Moreover, *PIF1* and *PIF3* also contain the active phytochrome A‐binding (APA) region, which is necessary for phytochrome A (phyA) binding (Leivar and Monte, 2014; Leivar and Quail, 2011; Shen *et al*., 2008). Until now, there has been little information on *PIFs* in crop. Six phytochrome‐interacting factor‐like (PIL) homologs (OsPIL11‐OsPIL16) have been found in rice by evaluating the rice databases (Nakamura *et al*., 2007). In addition, two other PIF proteins have been identified and characterized, including *LjPIF4* in *Lotus japonicas* (Ono *et al*., 2010) and *ZmPIF3* in maize (Gao *et al*., 2015; Kumar *et al*., 2016).

Activated phyB interacts with PIF1 and induces PIF1 phosphorylation and degradation under light irradiation (Krzymuski *et al*., 2014). It has been reported that phyB is able to induce tolerance to stresses by enhancing ABA sensitivity (Gonzalez *et al*., 2012; Staneloni *et al*., 2008). The phyB mutants were able to reduce water loss and improve drought tolerance by regulating stomatal opening and density (Boccalandro *et al*., 2009; Liu *et al*., 2012). In addition, *PIF1* can indirectly inhibit the gibberellic acid (GA) pathway and plays an important role in regulating the expression of ABA biosynthesis‐related genes and promoting ABA biosynthesis (Kim *et al*., 2008; Oh *et al*., 2009). *PIF1* also interacts with the ABA‐positive signalling component gene *ABI5*, which inhibits seed germination via ABA signalling (Kim *et al*., 2016). Thus, *PIF1* can regulate endogenous ABA and ABA signalling, and ABA can regulate plant adaptation to drought stress (Osakabe *et al*., 2014). However, the role of *PIF1* in drought stress remains poorly understood.

Although the regulatory functions of the PIFs have been widely explored in light responses, seed germination and hormone regulation, the roles of *PIFs* in the drought stress response remain elusive (Leivar and Quail, 2011; Oh *et al*., 2009). The rice PIF‐like protein OsPIL1 is down‐regulated under drought stress conditions (Todaka *et al*., 2012), and overexpression of both *DREB1A* and *OsPIL1* enhances drought tolerance in *Arabidopsis* (Kudo *et al*., 2016), while *ZmPIF3* regulates plant responses to drought stresses (Gao *et al*., 2015). Thus, PIFs likely play complicated roles under drought stress. In this study, we focused on the role of PIFs in water saving, drought resistance and grain yield and suggest that maize *ZmPIF1* is a promising candidate gene for transgenic breeding of water saving and drought‐resistant plants for crop improvement.

## Results

### Isolation and characterization of *ZmPIF1* encoding a phytochrome‐interacting factor in maize

**Figure 1:**
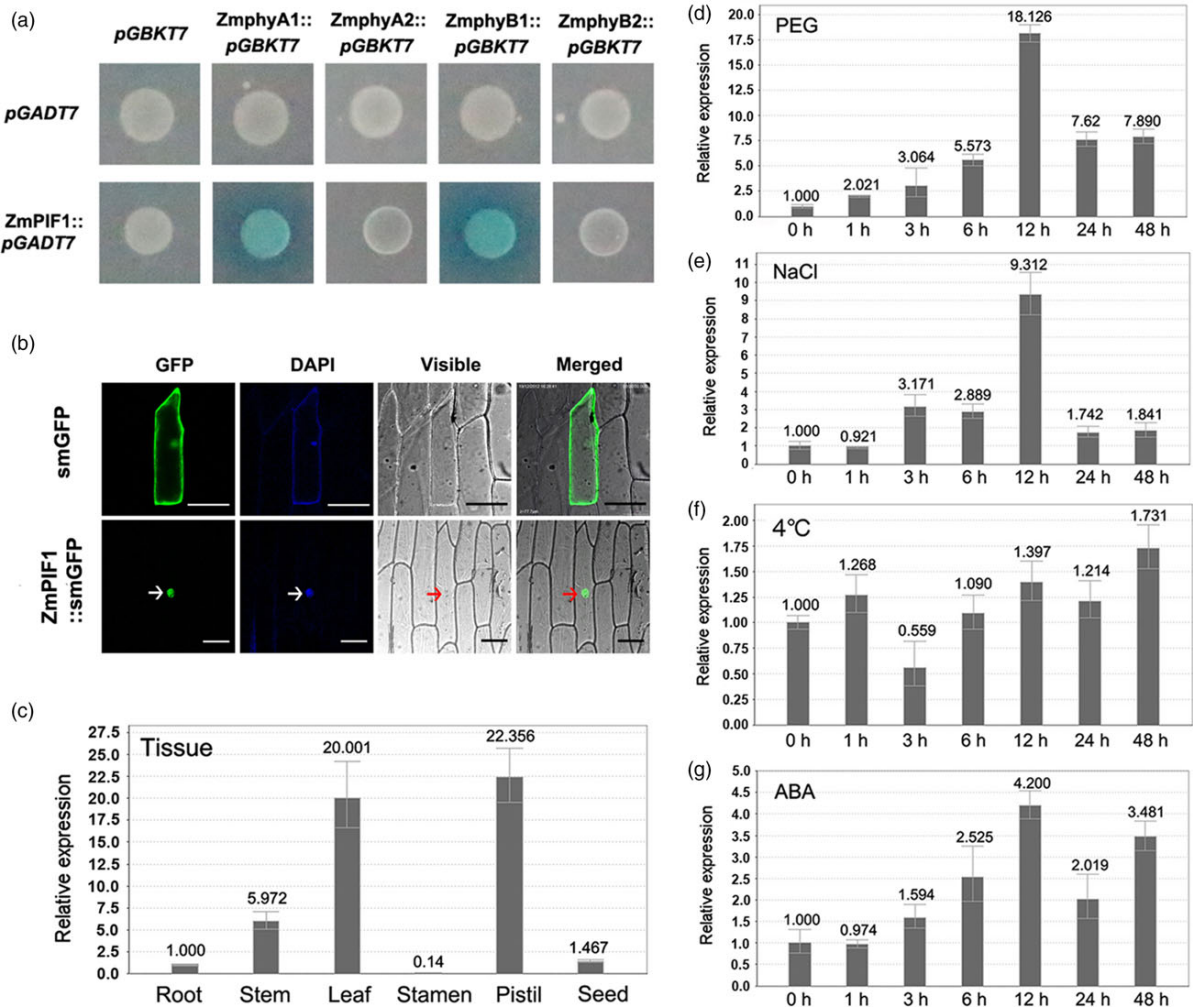
Y2H assays, subcellular localization and expression profiles of *ZmPIF1*. (a) Y2H assays. Positive interactions were determined using the auxotrophic selection media SD/‐Ade/‐His‐Leu/‐Trp and SD/‐Ade/‐His‐Leu/‐Trp+x‐α‐gal. pGADT7‐ZmPIF1 interacts with pGBKT7‐ZmPhyB and pGBKT7‐ZmPhyA. (b) Subcellular localization of *ZmPIF1*. GFP fusions of full‐length *ZmPIF1* were transiently transformed into onion epidermal cells. The plant nuclei were stained with DAPI. Images were obtained using confocal microscopy (GFP fluorescence, green; DAPI fluorescence, blue; visible, visible light image; merged, merged images of above three images). Empty vector (smGFP)‐transformed cells are shown as a control. Arrows indicate *ZmPIF1*‐localized nuclei. Bar = 100 mm. (c) Expression patterns of *ZmPIF1* in maize tissues at different developmental stages. (d‐g) Expression patterns of *ZmPIF1* under various abiotic stresses in seedlings (*n* = 20). (d) Drought stress (20% PEG6000); (e) salt stress (200 mm NaCl); (f) low temperature stress (4 °C); (g) abscisic acid (ABA) stress (100 μm). The relative expression was quantified by qRT‐PCR and normalized to β‐actin. Data represent the mean ± SD.

The clone of the target gene was 1704 bp in length, and a database search showed that the sequence was similar to those of other PIFs. Outside of the bHLH domain, the ZmPIF1 amino acid sequence also contained two characteristic domains, APB motif and APA motif (Figure S1). The Y2H study results indicated that the APB domain of ZmPIF1 interacts with maize phytochrome B1 (ZmPhyB1) and the APA domain of ZmPIF1 interacts with maize phytochrome A1 (ZmPhyA1) in yeast (Figure 1a). A phylogenetic analysis revealed that ZmPIF1 was closely related to ZmPIF3 in maize, PIF1 and PIF3 in *Arabidopsis*, and OsPIL15 and OsPIL16 in rice (Figure S2).

The full‐length *ZmPIF1* was introduced into the p2GWF7 vector, and a *ZmPIF1*‐green fluorescent protein (GFP) fusion protein was constructed to investigate the subcellular localization of ZmPIF1 (Gao *et al*., 2015). Transient expression assays suggested that the *ZmPIF1*‐GFP fusion protein was localized in the nucleus (Figure 1b), which was corroborated by bimolecular fluorescence complementation (BiFC) tests. The results suggest that *ZmPIF1* can interact with itself via BiFC and may form homodimers (Figure S3). Thus, *ZmPIF1* is located in the nucleus.

Different tissue expression patterns of *ZmPIF1* were examined. As shown in Figure 1, the expression of *ZmPIF1* was higher in pistils and leaves, while lower expression levels were observed in seeds, stamens and roots (Figure 1c). The expression level of *ZmPIF1* under polyethylene glycol (PEG), salt, ABA and low‐temperature treatments was examined and was significantly regulated by PEG, salt and ABA, while its expression did not change under cold temperature conditions (Figure 1d–g). Thus, *ZmPIF1* may play important roles in drought, salt and ABA responses.

### Expression of *ZmPIF1* enhances tolerance to drought stress in rice

The expression patterns suggest that *ZmPIF1* plays an important role in drought stress. To investigate whether the overexpression of *ZmPIF1* can improve drought resistance in rice, the full‐length of *ZmPIF1* was transformed into ‘Wuyunjing’ rice, and eleven transgenic lines were screened by qRT‐PCR. The qRT‐PCR results indicated that the expression level of *ZmPIF1* in transgenic plants could be detected, but not in the wild‐type (WT) or the vector control (VC) (Figure S4a). The *ZmPIF1* overexpression (OE) transgenic lines OE‐1, OE‐3 and OE‐7 were chosen for all assays in this study, and there were no obvious differences in plant morphology between the transgenic lines and the two controls under normal conditions (Figure S4b–d).

**Figure 2:**
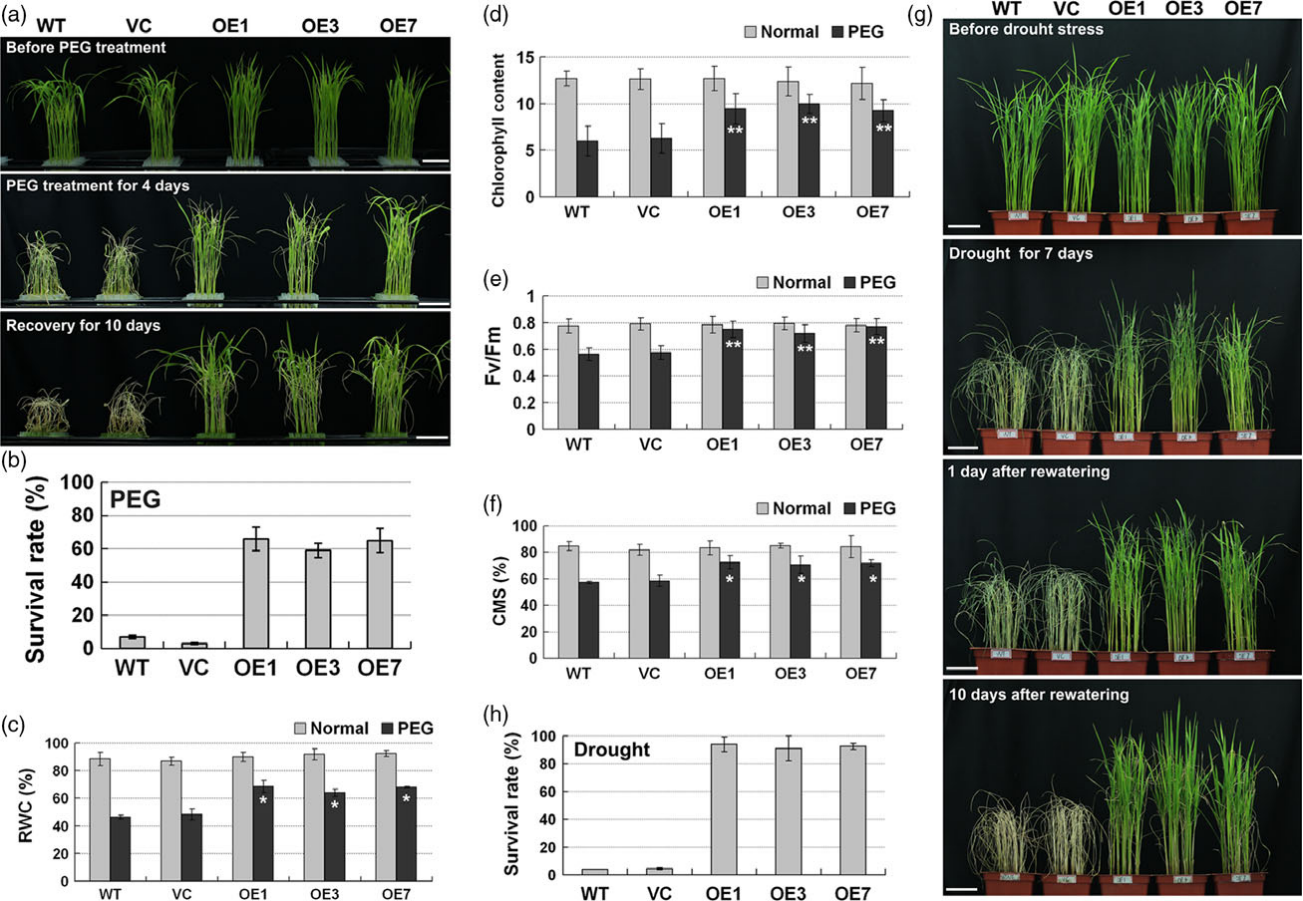
*ZmPIF1* improves drought tolerance of transgenic rice. (a) Seedlings treated with 20% polyethylene glycol (PEG) (*n* = 32). Two‐week‐old rice seedlings of *ZmPIF1* transgenic lines, wild‐type and vector controls were exposed to 20% PEG for 4 days and then allowed to recover for 10 days. Bar = 5 cm. (b) Survival rates of the transgenic and the control rice after PEG treatment. (c) Relative water content (RWC) (*n* = 20). (d) Chlorophyll content (*n* = 20). (e) Chlorophyll fluorescence (Fv/Fm) (*n* = 20). (f) Cell membrane stability (CMS) (*n* = 20). (c–f) Two‐week‐old rice seedlings were treated 20% PEG for 48 h. (g) *ZmPIF1* transgenic rice have enhanced tolerance to drought in soil. Forty‐day‐old seedlings of *ZmPIF1* transgenic rice, wild‐type and vector controls grown in soil subjected to drought stress for 7 days and then rewatered for 10 days. Bar = 5 cm. (h) Survival rates of transgenic and control rice after drought stress (*n* = 20–40). (b, h) Data represent the mean ± SD. (c–f) Data represent the mean ± SE. ** *t*‐test, with *P* < 0.01; *t‐test, with *P* < 0.05.

To investigate the drought tolerance of *ZmPIF1*, two controls and *ZmPIF1* transgenic rice were exposed to water deficit using a 20% PEG solution treatment. Under normal conditions, there were no differences in plant morphology between the two controls and *ZmPIF1* transgenic rice (Figure 2a). After 4 days of PEG treatment, the majority of the two control plants began to wither, while a few of the leaves in the transgenic rice were rolled and wilted (Figure 2a). After recovery in a normal hydroponic solution, some of the transgenic rice survived and recovered, while most of the leaves of the two control plants were still rolled and wilted (Figure 2a). Ten days after recovery, the survival rates of the two controls and the transgenic rice were determined. The survival rate of the transgenic lines, at ~60%, was higher than those of the two control plants, which had almost completely died (Figure 2b).

Four physiological parameters, relative water content (RWC), chlorophyll content, chlorophyll fluorescence and cell membrane stability (CMS), were assayed (Figure 2c–f). In this study, no differences were observed in these four physiological parameters between the two controls and the transgenic lines under nonstressed conditions. Under PEG treatment, the physiological parameters of the two controls were significantly lower than those of the *ZmPIF1* transgenic rice (Figure 2c–f). These results revealed that *ZmPIF1* improves water retention and photosynthetic and cell membrane stability capabilities compared with the controls under PEG treatment in rice.

To further verify the drought tolerance of *ZmPIF1*, two controls and *ZmPIF1* transgenic rice were exposed in soil to a water deficit. Before the drought treatment, the growth of two controls and transgenic rice was similar under normal conditions (Figure 2g, under normal conditions). After 7 days without watering, the leaves of the two control plants were rolled and wilted, while only a few leaves of the *ZmPIF1* transgenic rice had begun to roll and wilt (Figure 2g, drought for 7 days). After 1 day of recovery, the two control plants still had drought phenotypes. Moreover, more than half of the transgenic rice were green and healthy (Figure 2g, 1 day after rewatering). After 10 days of recovery in water, the majority of the two control lines did not recover and only approximately 5% survived, while more than 90% of the transgenic plants survived (Figure 2h). Taken together, these results reveal that *ZmPIF1* can increase drought tolerance in rice.

### Expression of *ZmPIF1* prevents water loss and participates positively in stomatal closure

**Figure 3:**
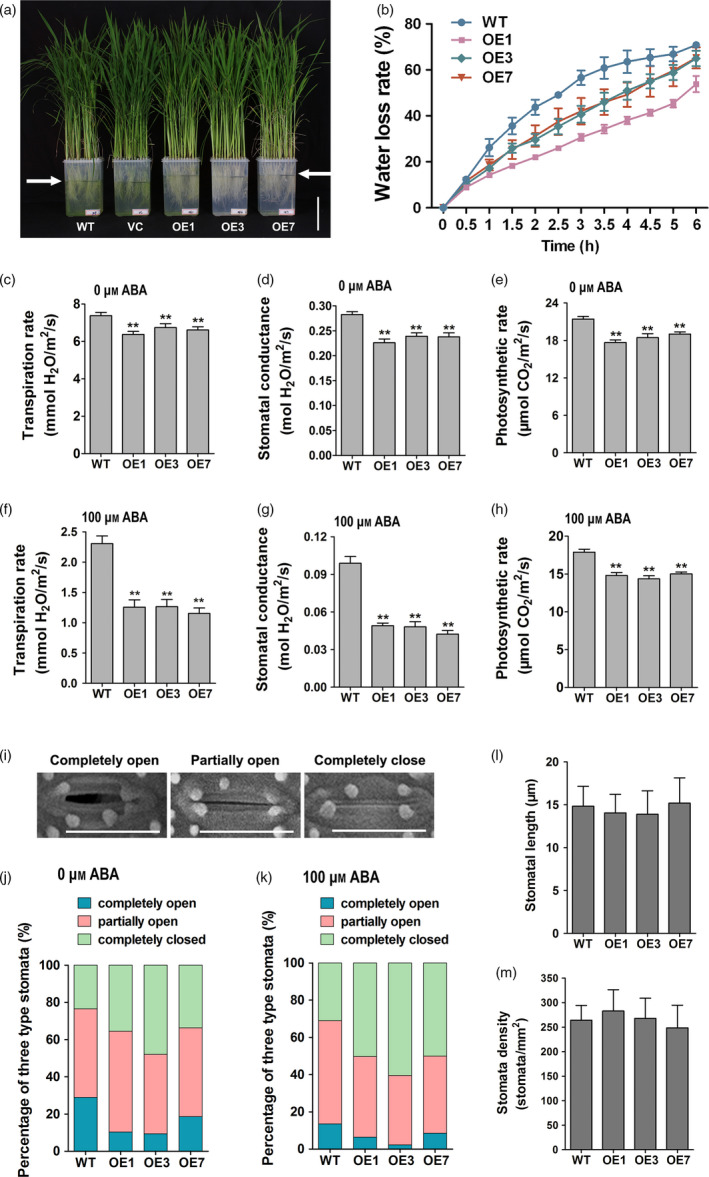
*ZmPIF1* enhanced stomatal closure and reduced transpiration in rice. (a) The phenotype of the *ZmPIF1* transgenic rice showed decreased transpiration. Seedlings of wild‐type, vector control and *ZmPIF1* transgenic rice cultured after 3 days; water level of *ZmPIF1* transgenic rice and control plants was marked with black lines. Bar = 10 cm. (b) Water loss assays for the leaves of the *ZmPIF1* transgenic lines and wild type were performed within 6 h (*n* = 5). (c–h) The transpiration rate, stomatal conductance and photosynthetic rate of the *ZmPIF1* transgenic lines and wild type: (c–e) no treatment and (f–h) 100 μm abscisic acid (ABA) treatment (*n* > 50). (i) Scanning electron microscopy images of three levels of stomatal opening. Bar = 20 μm. (j–k) The percentage of three levels of stomatal opening in *ZmPIF1* transgenic lines and wild type: (j) no treatment and (k) 100 μm ABA treatments (*n* > 400). All tests were performed in 40‐day‐old well‐watered plants in the glasshouse. Data represent the mean ± SE. **t‐test, with *P* < 0.01; *t‐test, with *P* < 0.05.

Interestingly, when *ZmPIF1* transgenic rice and WT rice were grown in different individual pots, the drought‐resistant phenotype of the *ZmPIF1* transgenic rice was more apparent compared with the WT phenotype. However, when the *ZmPIF1* transgenic and WT rice were planted in the same pot, the drought‐resistant phenotypes of the *ZmPIF1* transgenic and WT rice were not obviously different (data not shown). We hypothesize that *ZmPIF1* transgenic rice differs in terms of transpiration and may exhibit improved drought tolerance through water saving. To further investigate the involvement of *ZmPIF1* in modulating water saving, a water loss assay was tested. Thirty‐five well‐grown *ZmPIF1* transgenic rice and control seedlings, respectively, were transplanted into clear pots of the same size containing the same volume of hydroponic culture solution. After 3 days under normal conditions, the water levels of the *ZmPIF1* transgenic lines were obviously higher relative to those of the two controls (Figure 3a). These results demonstrate that *ZmPIF1* can significantly improve water retention in rice seedlings.

The water loss rates of *ZmPIF1* transgenic and WT rice were consistent with the water loss assay results. The leaves of *ZmPIF1* transgenic rice lost less water than the WT control (Figure 3b). Subsequently, we measured the transpiration rates and stomatal conductance levels of rice leaves. The transpiration rate and stomatal conductance levels of transgenic rice were lower relative to the WT control (Figure 3c,d). We speculated that overexpression of *ZmPIF1* in rice led to a lower rate of water loss.

In response to drought stress, stomatal movements are regulated to control water loss by transpiration (Murata *et al*., 2015). Thus, we measured the stomatal density, length and aperture in WT and *ZmPIF1* transgenic rice. In *ZmPIF1* transgenic rice, no significant alterations were observed in the density or length of stomata relative to WT rice (Figure 3l,m). More stomata were completely closed, and fewer stomata were completely open in the leaves of *ZmPIF1* transgenic lines compared with WT rice (Figure 3i,j). The aforementioned results indicate that the reduction in *ZmPIF1* stomatal aperture decreased the transpiration rate, which led to enhanced water saving and drought resistance in rice.

**Figure 4:**
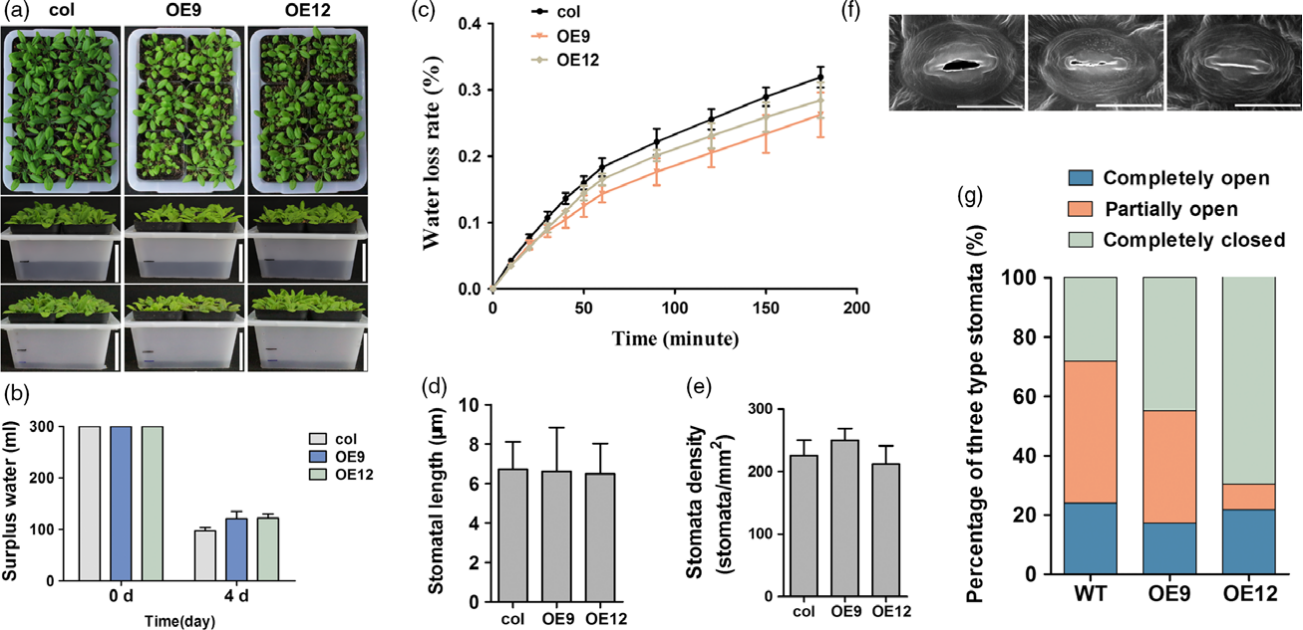
*ZmPIF1* enhanced stomatal closure and reduced transpiration in *Arabidopsis*. (a) The phenotype of *ZmPIF1* transgenic *Arabidopsis* and wild type (col) with reduced transpiration (*n* = 30). Seedlings of wild‐type (col) and *ZmPIF1* transgenic *Arabidopsis* were transplanted into the same transparent pots (containing the same weight of soil) and supplemented with 300 mL water after saturation of the soil water. After 4 days, the water level of *ZmPIF1* transgenic *Arabidopsis* and wild type (col) were marked with black lines. Bar = 5 cm. (b) Surplus water of wild‐type (col) and *ZmPIF1* transgenic *Arabidopsis* after 4 days. (c) Water loss assays for *ZmPIF1* transgenic *Arabidopsis*. Water loss assays for the leaves of the *ZmPIF1* transgenic lines and wild type (col) were performed within 200 min in *Arabidopsis* (*n* > 5). Comparisons of stomatal length (d) and stomatal density (e) in wild‐type (col) and transgenic *Arabidopsis* at the seedling stage (*n* > 200). (f) The stomatal aperture observed with a scanning electron microscope. Bar = 20 μm. (g) The percentage of three levels of stomatal opening in ZmPIF1 transgenic *Arabidopsis* and wild type (col) (*n* > 200). col: *Arabidopsis thaliana* L. Heynh, Columbia; OE9, OE12, two *ZmPIF1* transgenic lines.

To verify the function of *ZmPIF1*‐enhanced water saving and drought resistance in *Arabidopsis*, we constructed *ZmPIF1* overexpression transgenic plants of in *Arabidopsis*. Six T3 transgenic lines were obtained, and two of them were used to verify the function of *ZmPIF1* (Figure 4; Figure S5). A water loss assay was performed in *Arabidopsis*. Thirty well‐grown *ZmPIF1* overexpression *Arabidopsis* seedlings and WT seedlings were transferred into the same pot with 300 mL water. After 4 days, the water level was significantly higher in the pots containing the *ZmPIF1* transgenic *Arabidopsis* compared with the WT plants (Figure 4a,b). Measurements of the water loss rate from the leaves showed that *ZmPIF1* transgenic plants lost less water than WT plants (Figure 4c). Moreover, we measured the stomatal aperture, density and length of stomatal pores in the overexpression transgenic plants of *ZmPIF1* (Figure 4d–g), and the results were consistent with those in rice. The density and length of stomata showed no significant alterations in *ZmPIF1* transgenic *Arabidopsis* relative to in WT plants (Figure 4d–e). More stomata were completely closed and fewer stomata were completely open in the leaves of the *ZmPIF1* transgenic lines compared with WT *Arabidopsis* (Figure 4f–g). These results also suggested that enhanced water saving and drought resistance in *ZmPIF1* transgenic *Arabidopsis* were largely due to reducing stomatal opening.

### Expression of *ZmPIF1* increased ABA sensitivity in rice

Under drought stress, ABA promotes stomatal closure and decreases water loss (Desikan *et al*., 2004; Schroeder *et al*., 2001). Previous experiments have shown that ABA can induce the expression of *ZmPIF1* (Figure 1g); therefore, we speculated that *ZmPIF1* may be involved in the ABA pathway. In this study, we measured the transpiration rates and stomatal conductance levels of rice leaves treated with ABA. With ABA treatment, the transpiration rate and stomatal conductance levels of transgenic rice were significantly lower than that of the WT control (Figure 3f–h). We also compared the aperture of the stomata of WT and transgenic rice treated with ABA. The results showed that more stomata were completely closed and fewer stomata were completely open in the leaves of *ZmPIF1* transgenic rice compared with WT (Figure 3k). These results indicated that the transgenic rice potentially showed a hypersensitivity to ABA.

**Figure 5:**
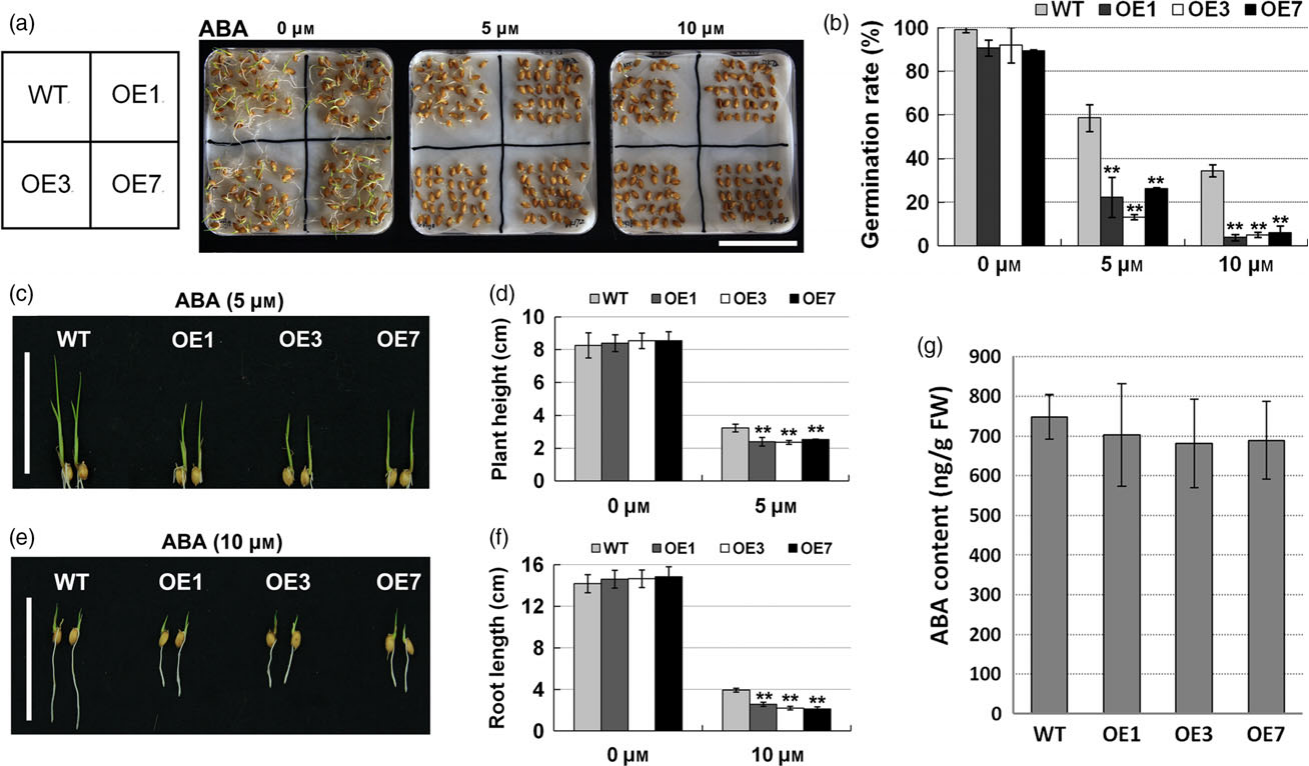
Increasing abscisic acid (ABA) sensitivity of *ZmPIF1* transgenic rice at the germination and seedling stages. (a) Germination phenotype of *ZmPIF1* transgenic and wild‐type rice seeds on wet filter paper containing 0, 5 or 10 μm ABA for 5 days (*n* = 35). Bar = 5 cm. (b) The germination rates of *ZmPIF1* transgenic and wild‐type rice seeds subjected to ABA treatment (*n* = 35). (c) Phenotype of plant height of *ZmPIF1* transgenic and wild‐type rice transplanted into water containing 5 μm ABA for 7 days (*n* = 20). Bar = 5 cm. (d) The plant height of the lines grown in normal and 5 μm ABA‐containing water for 7 days (*n* = 20). (e) Phenotype of the root length of *ZmPIF1* transgenic and wild‐type rice transplanted into water containing 10 μm ABA for 5 days (*n* = 20). Bar = 5 cm. (f) The root length of the lines grown in normal and 10 μm ABA‐containing water for 5 days (*n* = 20). (g) ABA content of *ZmPIF1* transgenic rice and wild type (*n* = 15). (b, g) Data represent the mean ± SD. (d, f) Data represent the mean ± SE. **t‐test, with *P* < 0.01; *t‐test, with *P* < 0.05.

To further verify whether the transgenic rice were sensitive to ABA, we measured the seed germination rates, root lengths and plant heights of WT and transgenic rice treated with ABA. In the absence of ABA, the seed germination rates of *ZmPIF1* transgenic lines and WT were not significantly different (Figure 5a,b). With increasing ABA concentrations, the germination rates of WT seeds decreased from 99% (0 μm ABA) to 59% (5 μm ABA) and 37% (10 μm ABA) (Figure 5b). However, the seeds of transgenic rice showed a hypersensitivity to ABA. Almost 90% of *ZmPIF1* transgenic rice seeds germinated with 0 μm ABA, but only 9% of the *ZmPIF1* transgenic rice germinated with 10 μm ABA (Figure 5a,b). Germination assays were also performed under mannitol and NaCl treatments. The same germination phenotypes of transgenic plants were shown in response to NaCl and mannitol (Figure S6). These results suggested that the transgenic rice seeds showed a hypersensitivity to ABA during the germination process.

For the root length and plant height measurements, the seedling growth rates of different genotypes were similar in the absence of ABA (Figure 5c,e). In response to different ABA concentrations (0, 5 and 10 μm), the root lengths and plant heights of the transgenic rice were more suppressed relative to those of WT (Figure 5c–f). These experiments demonstrated that root lengths and plant heights of transgenic rice showed a hypersensitivity to ABA during the postgermination process. These results indicated that *ZmPIF1* is a positive regulator of ABA signalling.

To more systematically evaluate the function of *ZmPIF1*, we analyzed the endogenous ABA levels in transgenic and WT rice, which were not significantly different under normal conditions (Figure 5g), suggesting that *ZmPIF1* is not involved in ABA synthesis but contributes significantly to the ABA signalling pathway.

### *ZmPIF1* affects the expression of ABA‐induced, stress‐responsive and stomata‐related genes

We performed a digital gene expression (DGE) analysis to determine the differential gene expression between WT and the transgenic rice at the seedling stage (Figure S7). Interestingly, the involvement of the hormone pathways, abiotic stress and stomatal‐related genes were modulated in leaves of *ZmPIF1*‐transgenic rice relative to WT (Table 1, Figure S8).

**Table 1:** Expression of plant hormone signal transduction, stress‐ and stomatal‐related genes identified by digital gene expression tag profiling

Gene ID	Description	Fold change
Plant hormone signal transduction		
LOC_Os01g64000	bZIP transcription factor, putative, expressed (OsABI5/OsbZIP10)	1.584962501
LOC_Os12g40920	bZIP transcription factor domain containing protein, expressed (bZIP88)	3.663107306
LOC_Os11g26760	Dehydrin, putative, expressed (OsRAB16C)	4.36923381
LOC_Os11g26790	Dehydrin, putative, expressed (OsRAB16A/OsRAB21)	2.584962501
LOC_Os11g47240	Leucine‐rich repeat receptor protein kinase EXS precursor, putative, expressed (BRI1)	2.571528352
LOC_Os06g48200	Glycosyl hydrolases family 16, putative, expressed (BRII)	3.335934953
LOC_Os02g43330	Homeobox associated leucine zipper, putative, expressed	1.972316441
LOC_Os04g54900	HLH transcription factor (OsILI1)	3.415037
LOC_Os11g39000	Helix‐loop‐helix DNA‐binding domain containing protein, expressed (OsILI2)	4.196397
LOC_Os10g02880	O‐methyltransferase, putative, expressed	3.832890014
LOC_Os01g24710	Jacalin‐like lectin domain containing protein, expressed (SalT)	3.310787537
LOC_Os01g06560	Transcription factor HBP‐1b, putative, expressed	4.087462841
LOC_Os08g31340	Heavy metal‐associated domain containing protein, expressed	1.846087317
LOC_Os03g18490	RPGR, putative, expressed	2.874469118
LOC_Os07g03730	SCP‐like extracellular protein, expressed	‐3.115538327
Stress‐related genes		
LOC_Os10g36180	Expressed protein (OsRD29)	2.662965013
LOC_Os11g26780	Dehydrin, putative, expressed (OsRab16D)	3.841302254
LOC_Os11g26790	Dehydrin, putative, expressed (OsRAB16A/OsRAB21)	2.584962501
LOC_Os11g26750	Dehydrin, putative, expressed (OsRAB16B)	2.938599455
LOC_Os01g50910	Late embryogenesis abundant protein, group 3, putative, expressed (OsLEA14a)	1.767165832
LOC_Os11g37960	WIP4—Wound‐induced protein precursor, expressed (OsPR4b)	2.52118263
LOC_Os11g37950	WIP3—Wound‐induced protein precursor, expressed (OsPR4c)	4.377933505
LOC_Os07g48010	Peroxidase precursor, putative, expressed (POX8_1)	1.674759171
LOC_Os07g02440	Peroxidase precursor, putative, expressed (OsPOD1)	1.722466024
LOC_Os01g51990	AN1‐like zinc finger domain containing protein, expressed (OsSAP13)	‐4.523561956
LOC_Os09g35030	Dehydration‐responsive element‐binding protein, putative, expressed (OsDREB1A)	‐2.767914182
LOC_Os09g35010	Dehydration‐responsive element‐binding protein, putative, expressed (OsDREB1B)	‐3.040157126
LOC_Os08g43334	HSF‐type DNA‐binding domain containing protein, expressed (OsHsfB2b)	2.130629443
LOC_Os04g14680	OsAPx3—Peroxisomal Ascorbate Peroxidase encoding gene 5,8, expressed (ROS‐related genes)	1.628031223
Stomatal‐related gene		
LOC_Os01g60770	Expansin precursor, putative, expressed (OsEXPA2)	2.444048586
LOC_Os05g39990	Expansin precursor, putative, expressed (OsEXPA4)	1.90442234
LOC_Os10g40710	Expansin precursor, putative, expressed (OsEXPB2)	1.865982652
LOC_Os10g40720	Expansin precursor, putative, expressed (OsEXPB3)	3.759333407
LOC_Os10g40730	Expansin precursor, putative, expressed (OsEXPB4)	4.544320516
LOC_Os10g40700	Expansin precursor, putative, expressed (OsEXPB6)	2.911463325
LOC_Os03g01270	Expansin precursor, putative, expressed (OsEXPB7)	1.959358016
LOC_Os02g44108	Expansin precursor, putative, expressed (OsEXPB11)	2.637429921
LOC_Os02g40240	Receptor kinase, putative, expressed (LP2)	‐2.321928095
LOC_Os10g40090	Expansin precursor, putative, expressed (OsEXPB9)	‐1.736965594
LOC_Os01g68598	Expressed protein (EPFL9)	‐1.823122238
LOC_Os11g32100	Inducer of CBF expression 1, putative, expressed, (OsSCRM1)	2.280107919

Selected up‐regulated and down‐regulated genes in *ZmPIF1* transgenic rice relative to wild‐type plants. Genes with at least a 1.5‐fold change in the *ZmPIF1* transgenic rice are shown.

The expression levels of genes involved in the ABA signalling network were altered in *ZmPIF1* transgenic rice compared with WT plants. The expression levels of *OsABI5* and *OsbZIP88*, which were predicted to be ABA‐responsive binding factors, were elevated in *ZmPIF1* transgenic rice (Table 1). The expression levels of *OsRAB16A* and *OsRAB16C*, which have been reported to be the downstream of ABA signalling (Hong *et al*., 2009; Wang *et al*., 2007), were increased in *ZmPIF1* transgenic rice (Table 1). However, the expression levels of *OsNCED1*‐*5*, which are the key genes in ABA biosynthesis (Zhu *et al*., 2009), did not significantly change in *ZmPIF1* transgenic and WT rice (data not shown).

The transcript levels of many well‐known drought resistance‐related genes, including *OsRAB16A*,* OsRAB16B*,* OsRAB16D*,* OsPR4b* and *OsPR4c*, were up‐regulated in *ZmPIF1* transgenic rice compared with WT (Table 1) (Du *et al*., 2010; Wang *et al*., 2007, 2011). Overexpression of *Leaf Panicle 2* (*LP2*), which is a leucine‐rich repeat receptor‐like kinase, leads to a decrease in stomatal closure (Wu *et al*., 2015). In the present study, the *LP2* expression level was decreased in *ZmPIF1* transgenic rice. To investigate the differentially expressed genes, some *EXPANSIN* family genes related to stomata aperture were identified. The expression levels of these genes, including *OsEXPA2*,* OsEXPA4*,* OsEXPB2*,* OsEXPB3*,* OsEXPB4*,* OsEXPB6*,* OsEXPB7* and *OsEXPB11*, were up‐regulated at least 1.5‐fold (Table 1) (Liu *et al*., 2012).

### *ZmPIF1* increases the grain yield in rice

**Figure 6:**
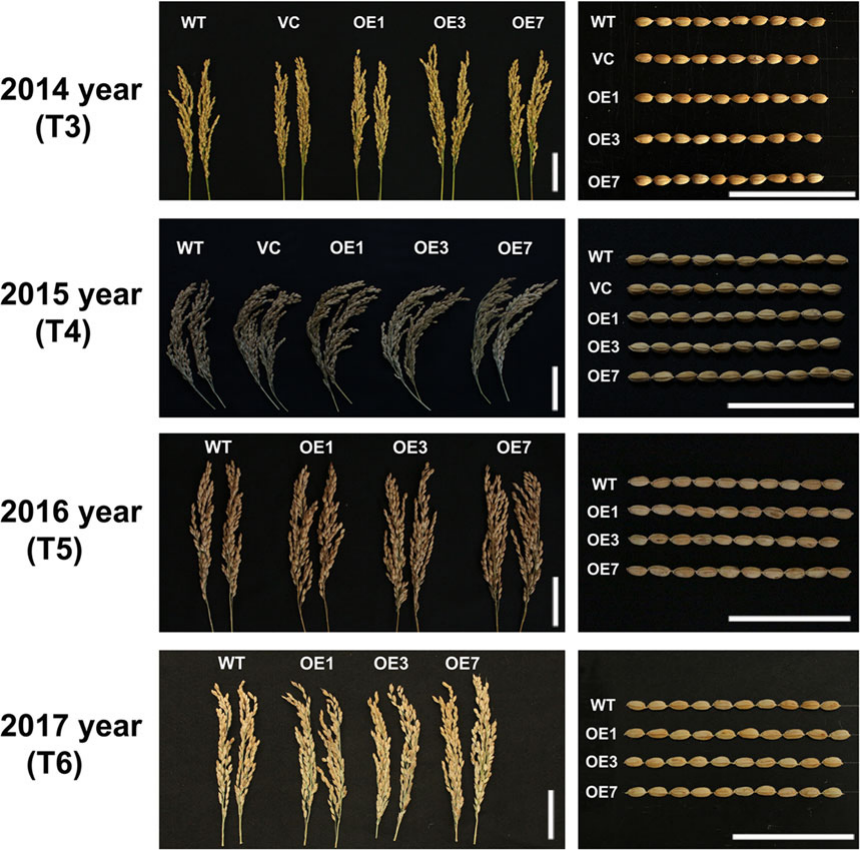
Panicle phenotype and seed morphology of *ZmPIF1* transgenic and wild‐type rice. Agronomic traits of the *ZmPIF1* transgenic rice under normal conditions for three cultivating seasons (2014, 2015, 2016 and 2017). Bar = 5 cm.

**Figure 7:**
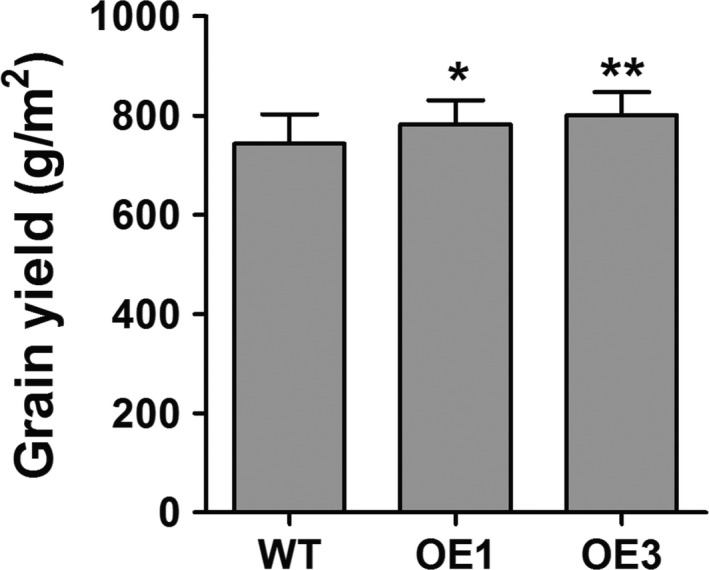
The unit area yield of *ZmPIF1* transgenic and wild‐type rice in 2017. The planting density was 30/m^2^. The unit area yields were calculated for four replicates in different regions and >100 plants per replicate. Data represent the mean ± SE. **t‐test, with *P* < 0.01; *t‐test, with *P* < 0.05.

Grain yield is the one of most important agronomic traits of crops. Thus, we examined several agronomic traits of WT and *ZmPIF1* transgenic rice over 4 years of cultivation (2014, 2015, 2016 and 2017). The 4‐year data sets were consistent, but the number of scored plants was reduced in 2014, while the data for 2015, 2016 and 2017 showed greater statistical rigour (Table 2; Table S1). In 2015, 2016 and 2017, *ZmPIF1* transgenic rice showed an increased grain yield relative to WT because of the increased numbers of tillers and panicles (Table 2; Figure 6; Table S1). Moreover, the unit area yield of *ZmPIF1* transgenic lines was higher than in the WT control in 2017 (Figure 7). An additional interesting phenotype of *ZmPIF1* transgenic plants was that their tiller angles were significantly wider than those of WT plants (Figure S9). It is speculated that *ZmPIF1* is important for grain yield production by increasing the numbers of panicles.

**Table 2:** Agronomic traits of *ZmPIF1* transgenic rice grown in paddy field conditions in 2015, 2016 and 2017

Lines	No. of tillers per plant	Panicle number per plant	Panicle length (cm)	No. of grains per panicle	Filled grains per panicle	Seed‐setting rate (%)	1000‐Grain weight (g)	Grain yield per plant (g)
2015								
WT	8.96 ± 1.97	8.92 ± 2.00	15.16 ± 1.93	152.20 ± 28.57	136.70 ± 25.12	90.32 ± 1.79	25.47 ± 0.23	20.70 ± 3.00
VC	10.04 ± 2.49	9.96 ± 2.56	15.58 ± 1.60	160.40 ± 15.74	134.40 ± 10.43	83.10 ± 4.34**	26.55 ± 0.25**	20.11 ± 2.97
*ZmPIF1*								
OE1	13.87 ± 2.75**	13.83 ± 2.74**	15.63 ± 1.68*	145.60 ± 24.77	120.00 ± 26.70	81.61 ± 2.79**	27.54 ± 0.05**	27.08 ± 3.10*
OE3	13.75 ± 2.98**	13.54 ± 3.15**	15.27 ± 2.27	166.60 ± 18.64	122.80 ± 14.10	75.94 ± 4.74**	25.99 ± 0.13**	25.83 ± 3.06
OE7	13.17 ± 3.35**	12.17 ± 2.85**	15.87 ± 1.90**	167.00 ± 25.62	124.70 ± 19.28	73.62 ± 5.81**	25.17 ± 0.18*	25.59 ± 4.35
2016								
WT	10.53 ± 1.38	10.53 ± 1.38	17.28 ± 0.25	141.43 ± 9.59	134.00 ± 9.86	95.09 ± 3.20	24.25 ± 0.18	29.07 ± 5.33
*ZmPIF1*								
OE1	12.60 ± 2.28**	12.53 ± 2.33**	16.44 ± 0.23**	138.27 ± 10.03	128.33 ± 9.90	93.06 ± 4.52**	28.66 ± 0.04**	35.82 ± 8.61
OE3	13.80 ± 2.44**	13.73 ± 2.55**	16.49 ± 0.30**	148.07 ± 12.76*	136.97 ± 12.87	92.35 ± 4.56**	27.41 ± 0.08**	38.44 ± 7.93*
OE7	13.60 ± 1.90**	12.63 ± 1.88**	16.71 ± 0.66**	145.97 ± 13.87	134.37 ± 17.05	92.00 ± 8.36**	25.57 ± 0.14**	30.73 ± 6.96
2017								
WT	12.34 ± 2.36	12.13 ± 2.45	15.13 ± 1.52	91.00 ± 10.08	80.44 ± 12.05	89.98 ± 4.58	27.33 ± 0.11	24.78 ± 1.99
*ZmPIF1*								
OE1	14.01 ± 3.12*	13.62 ± 2.25*	14.83 ± 2.04	87.24 ± 12.63	71.12 ± 10.71*	85.23 ± 4.86*	30.39 ± 0.12**	25.63 ± 1.61*
OE3	14.13 ± 3.13**	14.07 ± 2.22**	14.27 ± 2.27	106.34 ± 18.26**	80.59 ± 22.03	80.91 ± 7.13**	27.81 ± 0.06**	26.69 ± 1.56**
OE7	13.35 ± 2.59*	13.04 ± 2.59*	14.51 ± 1.52	102.12 ± 16.82*	84.45 ± 14.11	83.38 ± 6.16**	27.43 ± 0.11	25.00 ± 2.28

Values are the mean ± SD (*n* > 15). * and ** indicate significant differences at *P* < 0.05 and *P* < 0.01, respectively.

## Discussion

### *ZmPIF1* plays a positive role in drought tolerance

The overexpression of some stress‐related genes can improve the environmental stress tolerance of plants (Tang *et al*., 2012). As previously discussed, the expression of *ZmPIF1* was strongly up‐regulated by PEG and salt stress (Figure 1d,e). Although PIFs have recently attracted much interest, their functions in abiotic stress remain largely unexplored. Some data have shown that *PIF4* expression may be regulated by high temperature in *Arabidopsis* (Franklin *et al*., 2011; Koini *et al*., 2009; Stavang *et al*., 2009). Todaka *et al*. (2012) reported that drought down‐regulates the PIF‐like gene *OsPIL1*. In our previous study, the maize PIF gene *ZmPIF3*, a homolog of *ZmPIF1*, was found to function in the response to abiotic stress (Gao *et al*., 2015). Following exposure to drought stress, the survival rates of the *ZmPIF1* transgenic rice were higher than in the two control plants grown in a hydroponic solution or soil (Figure 2). The RWC, chlorophyll content, chlorophyll fluorescence, and CMS of the *ZmPIF1* transgenic rice were significantly increased under stress conditions. These results suggested that PIFs may be involved in regulating plant growth and development, as well as in responding to drought stress.

### *ZmPIF1* positively regulates ABA‐dependent drought tolerance by regulating the stomata aperture

The drought responses of plants are complicated and modulated by multiple molecular pathways. Stomatal pores are involved in drought tolerance (Hetherington and Woodward, 2003; Schroeder *et al*., 2001). Stomatal responses can regulate water loss from plants to control the utilization of water (Murata *et al*., 2015). The *ZmPIF1* transgenic rice and *Arabidopsis* all showed lower water loss rate than WT (Figure 3a,b; Figure 4a–c) and significantly promoted stomatal closure and a reduced stomatal conductance and transpiration rate (Figure 3c–e, i,j; Figure 4f,g). Consequently, *ZmPIF1* transgenic plants could better avoid dehydration when the soil water became limiting in both rice and *Arabidopsis*. These findings indicate that *ZmPIF1* plays an important role in water saving by regulating the stomatal aperture.

Plants regulate gene expression levels and stomatal apertures in response to drought stress mainly by enhancing ABA biosynthesis and signalling (Cutler *et al*., 2010; Pizzio *et al*., 2013). As previously discussed, the *ZmPIF1* expression level was rapidly induced by ABA (Figure 1g), and *ZmPIF1* transgenic rice had higher survival rates after exposure to drought stress, which promoted stomatal closure (Figure 2; Figure 3c–e,j). Therefore, further exploration of the relationship between ABA and *ZmPIF1* was essential. *ZmPIF1* transgenic rice were tested for ABA responsiveness, and they exhibited a hypersensitive to ABA during germination and postgermination processes and in the seedling stage (Figure 3f–h, k; Figure 5). Moreover, the endogenous ABA levels in transgenic rice were also tested under normal conditions (Figure 5g). However, the endogenous ABA level of *ZmPIF1* transgenic rice was not significantly altered. Thus, *ZmPIF1* was not involved in ABA biosynthesis. We hypothesized that *ZmPIF1* participates in the ABA signalling pathway to reduce the stomatal aperture and inhibit the transpiration rate, thereby enhancing water saving and drought‐tolerance in the transgenic rice.

Digital gene expression profiling can assist in screening for key genes and possibly mechanisms. Under normal conditions, the DGE profiles of the *ZmPIF1* transgenic and WT control rice were compared to identify differentially expressed genes. The expression levels of the ABA biosynthesis‐related genes *OsNCED1*‐*5* were not significantly changed in *ZmPIF1* transgenic or WT rice (Zhu *et al*., 2009), consistent with the endogenous ABA content (Figure 5g), which further suggested that *ZmPIF1* was not involved in the ABA biosynthesis pathway.

*OsABI5* is a rice bZIP transcription factor, and *OsABI5*‐overexpression rice are hypersensitive to ABA. In *Arabidopsis*, in contrast, the ABA sensitivity of abi5‐1 can be recovered by *OsABI5* in a complementation test (Zou *et al*., 2008). *OsbZIP88* is also a bZIP transcription factor that can interact with *OsbZIP71* by forming heterodimers, while *OsbZIP71* may be involved in salt and drought tolerance (Liu *et al*., 2014). Relative to WT plants, the expression levels of *OsABI5* and *OsbZIP88* increased in *ZmPIF1* transgenic rice. Moreover, the expression levels of ABA signalling downstream genes *RAB16A* and *RAB16C* increased in *ZmPIF1* transgenic rice (Table 1; Hong *et al*., 2009; Wang *et al*., 2007). These results suggested that *ZmPIF1* participated downstream in the ABA signalling pathway through bZIP transcription factors to positively regulate the ABA signalling pathway.

*EXPANSIN* is a cell wall protein that is involved in regulating the stomatal aperture and stomatal density (Lü *et al*., 2013; Marowa *et al*., 2016; Wei *et al*., 2011; Zhang *et al*., 2011). Our DGE results revealed that eight *EXPANSIN* genes were up‐regulated in *ZmPIF1*‐transgenetic rice (Table 1). The expression of *LP2*, a leucine‐rich repeat receptor‐like kinase, was decreased in *ZmPIF1* transgenic rice. The expression of *LP2* is down‐regulated by drought, while transgenic plants overexpressing *LP2* have increased stomatal apertures in leaves (Wu *et al*., 2015). The expression levels of stomatal‐related genes also revealed that *ZmPIF1* might participate in regulating the stomatal aperture. In addition, *ZmPIF1* transgenic lines showed an induction of the expression of stress‐related genes, such as *OsRAB16A*,* OsRAB16B*,* OsRAB16D*,* OsPR4b* and *OsPR4c*, even under normal condition (Table 1; Du *et al*., 2010; Wang *et al*., 2007). Taken together, the DGE analysis indicated that *ZmPIF1* promoted closure of stomata and decreases in the transpiration rate by influencing the ABA‐dependent signalling pathway, which enhances water saving and drought resistance in rice.

### *ZmPIF1* transgenic rice have an increased grain yield

In crops, overexpression of stress tolerance genes may be lead to abnormal development and productivity loss (Dubouzet *et al*., 2003; Nakashima *et al*., 2007; Yu *et al*., 2013). Thus, it is also important to develop transgenic plants that can enhance stress tolerance and maintain grain yields. Under normal conditions, *ZmPIF1* can enhance grain yields in transgenic rice. The increased grain yield was mainly attributable to the increase in the number of panicles, without a substantial change in the number of spikelets per panicle (Table 2; Figure 6; Figure 7; Table S1). An additional phenotype of *ZmPIF1*‐transgenetic rice was their tiller angle, which was significantly wider than that of WT rice (Figure S9). Thus, the wider tiller angle of *ZmPIF1* transgenic rice provided a larger growth space, promoting increased growth of the tillers relative to the WT rice. The grain yields were increased in *ZmPIF1* transgenic lines, indicating a wider applicability of *ZmP*IF1 for crop improvement.

PIFs function as a signal hub in cells and participate in multiple signalling pathways. To date, many studies have shown that PIFs can affect Brassinosteroid (BR; de Lucas and Prat, 2014; Oh *et al*., 2012; Zhang *et al*., 2009). BR is associated with the plant panicle, and BRs are involved in panicle development in rice. Some BR signal transgenic plants appear to have more tillers, larger panicles, and more seeds per panicle than wild‐type plants (Mori *et al*., 2002; Wu *et al*., 2008; Zhang *et al*., 2009). In the present study, the DGE results showed that BR signalling pathway genes, such as BRI1 (LOC_Os11g47240), BRII (LOC_Os06g48200), OsILI1 (LOC_Os04g54900) and OsILI2 (LOC_Os11g39000), were significantly induced in *ZmPIF1* transgenic lines (Table 1). Therefore, we hypothesized that *ZmPIF1* might affect number of panicles by influencing other signalling pathways such as BR.

In this study, *ZmPIF1* was found to be a positive regulator of ABA signalling and to enhance water saving and drought resistance by reducing stomatal opening to control water loss. *ZmPIF1* can enhance drought tolerance and improve the grain yield of rice, which indicates that *ZmPIF1* plays important roles in drought tolerance and crop improvement. Further investigations are necessary to establish how *ZmPIF1* responds to drought stress with ABA signalling‐induced stomatal closure.

## Experimental procedures

### Plant materials and stress treatments

Maize ‘Zhengdan 958’ (*Zea mays*, hybrid line) was germinated for 6 days. The seedlings were transferred to hydroponic growth conditions at 25 °C with a 16/8‐h photoperiod. Four‐leaf‐stage maize seedlings were used for all the stress treatments. For the ABA treatment, twenty well‐grown seedlings were treated for 0, 1, 3, 6, 12, 24 and 48 h with 100 μm ABA (*n* = 20). For high‐salinity and PEG treatment, twenty well‐grown seedlings were treated for 0, 1, 3, 6, 12, 24 and 48 h with 200 mm NaCl or 20% PEG6000 (*n* = 20). For the cold treatment, twenty well‐grown seedlings were treated for 0, 1, 3, 6, 12, 24 and 48 h in a growth chamber at 4 °C for cold stress. (*n* = 20). All tests were repeated a minimum of three times.

Maize plants were grown in the field to obtain different tissues. At the jointing stage, total RNA from roots, stems and leaves was isolated separately. Stamens and pistils were sampled at heading stage.

The wild‐type rice material ‘Wuyunjing’ (*Oryza sativa* L.) and transgenic rice were grown in a chamber at 28/25 °C with a 16/8‐h light/dark cycle and 70% relative humidity. The wild‐type *Arabidopsis thaliana* (Columbia 0 type) and transgenic *Arabidopsis* were placed in a climate chamber at 22 °C with 70% relative humidity and a 12‐h light/12‐h dark photoperiod. The growth and harvesting of transgenic lines were performed under the same conditions. All tests were repeated a minimum of three times.

### Sequence homology and phylogenetic analyses

Based on the *PIF3* and *PIF1* gene sequences in *Arabidopsis*, a maize PIF gene, named *ZmPIF1*, was obtained from GenBank and MaizeGDB. The sequence of *ZmPIF1* was analyzed at the websites (http://www.expasy.org/, http://www.plantgdb.org/, http://www.ncbi.nlm.nih.gov/ and http://www.maizegdb.org/). The phylogenetic analysis was generated with DNAMAN and MEGA version 6.

### Yeast two‐hybrid (Y2H) assays

A kit for two‐hybrid analysis was obtained (Oebiotech, Shanghai, China). This kit contained all the tools essential for two‐hybrid assay, including vectors: pGBKT7, providing the GAL4 DNA‐binding domain, and pGADT7, providing the GAL4 activation domain. The following plasmids were constructed in this study: pGBKT7‐ZmPhyA1, pGBKT7‐ZmPhyA2, pGBKT7‐ZmPhyB1, pGBKT7‐ZmPhyB2 and pGADT7‐ZmPIF1.

### Subcellular localization

*ZmPIF1* was fused upstream of the GFP gene in the p2GWF7 expression vector and transformed into living onion epidermal cells by biolistic bombardment with a GeneGu. The subcellular location of ZmPIF1 was measured as stated in Gao *et al*. (2015).

### Gene expression quantified by qRT‐PCR

Total RNAs were extracted using the manufacturer's instructions of the TRIzol reagent (Takara, China), and reverse transcription reactions were performed using manufacturer's instructions of the Transcriptor First Strand cDNA Synthesis kit (Roche, Mannheim, IN, Germany). qRT‐PCR was performed using a SYBR Green Master Mix kit (Roche, Mannheim, IN, Germany) and specific primers (Table S2) on an ABI 7300 system. Three separate biological replicates were carried out for the qRT‐PCR experiments.

### Rice and *Arabidopsis* transformation

To create overexpression constructs of *ZmPIF1*, a fragment of *ZmPIF1* was cloned in maize. The *ZmPIF1* fragment was then inserted into the binary plasmid p1011, which contains a ubiquitin promoter. This construct was introduced into *Agrobacterium tumefaciens* strain EHA105, and transgenic rice of *ZmPIF1* were produced as described by Xu *et al*. (2005).

The *ZmPIF1* fragment was subcloned into the vector LZ007, in which transgene expression is under control of the CaMV 35S promoter. Transformation of *Arabidopsis* was performed by the floral dip method (Clough and Bent, 1998) using *Agrobacterium tumefaciens* strain GV3101.

### Drought phenotype analysis

For PEG treatment at the seedling stage, 2‐week‐old T3 *ZmPIF1* transgenic and two control rice were treated for 4 days with 20% PEG. Ten days after recovery, the survival rate of each line was measured. For drought treatment in soil, 40‐day‐old rice from each line were sown in individual pots. Water was withheld from the plants of each line for 7 days and then recovered. After 10 days of recovery in water, the survival rate of each line was determined.

### Physiological measurements

The chlorophyll florescence, chlorophyll content, RWC and CMS values were measured as stated in Gao *et al*. (2015). All of the tests were measured under normal or stress conditions (20% PEG6000 for 2 days). These tests used 2‐week‐old rice of each line.

### Phenotype of transpiration assay

Hydroponic cultured seedlings of T3 *ZmPIF1* transgenic rice and two controls (40 days old) were transplanted into the same transparent pot filled with hydroponic culture solution. Thirty‐five seedlings of each line were planted in each pot and grown under normal conditions. After 3 days, the water level of each line was marked with black lines.

Thirty‐day‐old seedlings of WT (col) and T3 *ZmPIF1* transgenic *Arabidopsis* were transplanted into the same transparent pots (containing the same weight of soil) with addition of 300 mL water after saturation of the soil water. After 4 days, the water levels of *ZmPIF1* transgenic *Arabidopsis* and WT were marked with black lines. The remaining water in the WT and *ZmPIF1* transgenic *Arabidopsis* was measured after 4 days.

### Measurements of water loss rate, transpiration rate and stomatal conductance

Leaves of 40‐day‐old rice of *ZmPIF1* transgenic lines and WT were detached. Leaves of 30‐day‐old *Arabidopsis* of *ZmPIF1* transgenic lines and WT were detached. The water loss rates of each line were calculated. A portable photosynthesis system (Li‐Cor 6400; Li‐Cor, Lincoln, NE) was used to test the stomatal conductance and transpiration rates in rice. Flag leaves of 40‐day‐old rice of each line were measured. For ABA treatment, thirty 40‐day rice seedlings were treated for 3 h with 100 μm ABA (*n* = 40).

### Scanning electron microscopy (SEM) images of stomata

Flag leaves of 40‐day‐old rice of *ZmPIF1* transgenic lines and WT were detached. Leaves of 30‐day‐old *Arabidopsis* of *ZmPIF1* transgenic lines and WT were detached and fixed in glutaraldehyde (2.5%), and images of the stomata were obtained by environmental scanning electron microscopy (XL‐30ESEM, PHILIPS, Netherland). Stomatal densities, lengths and apertures were measured randomly using Image‐Pro Plus6.0 software (Media Cybernetics, USA).

### Germination assay and growth measurement

Thirty‐five seeds were placed in square dishes supplemented with 0, 5 and 10 μm of ABA after geminating for 1 day. After 5 days, the germination rate of each line was determined. Additionally, twenty seeds were transplanted into 96‐well plates in which the bottoms were moved after the shoots reached 2 cm. Seeds were grown in water containing 0 and 5 μm ABA. After 7 days of growth, plant heights were measured. Concurrently, twenty seeds were grown in water containing 0, 10 μm ABA. After 5 days, root lengths were measured.

### Quantification of the endogenous ABA contents

The ABA levels of *ZmPIF1* transgenic lines and the WT were quantified as stated in Zhang *et al*. (2015). Flag leaves 40‐day‐old plants from each line were harvested to measure the endogenous ABA contents.

### DGE analysis

Thirty well‐grown WT and *ZmPIF1* transgenic plant seedlings each were selected. The second fully expanded leaves of WT and ZmPIF1 transgenic seedlings were rapidly cut, frozen and stored. We established three replicates of the WT control and named them WT‐1, WT‐2 and WT‐3. We also established OE1, OE3 and OE7 as three replicates of *ZmPIF1* transgenic plants and designated OE1, OE3 and OE7 as F1‐1, F1‐2 and F1‐3. Total RNAs were extracted in accordance with the instructions provided with the RNAprep pure Plant Kit (Tiangen, China). DGE was performed in accordance with the standard protocol of Beijing Genomics Institute (http://www.genomics.cn/index; Shenzhen, China).

### Grain yield analysis

Thirty‐day‐old seedlings of *ZmPIF1* and WT control were transplanted in a paddy field in Jiangsu Province, China. Agronomic traits were calculated for three replicates and 3 (2014), 20 (2015), 15 (2016) and 40 (2016) plants per replicate using statistical analyses. The unit area yields of *ZmPIF1* transgenic and WT rice in the field were tested in 2017. The planting density was 30/m^2^. The unit area yields of ZmPIF1 transgenic and WT rice were calculated for 4 replicates in different regions and >100 plants per replicate.

## Accession numbers

Sequence data for the genes described in this article can be found in the Maize Genome Initiative or GenBank/EMBL databases under following accession numbers: ZmPIF1 (GRMZM2G115960_T03), ZmPIF3 (GRMZM2G387528_T02), PIF1 (Q8GZM7.1) and PIF3 (Q80536.1).

## Conflict of interest

The authors declare no conflict of interest.

## Supporting information


**Figure S1** Sequence alignment of *ZmPIF1* in other *Zea mays* and *Arabidopsis* PIFs family.
**Figure S2** Phylogenetic tree analysis of *ZmPIF1* from other PIFs family.
**Figure S3** Confirmation of the subcellular localization of *ZmPIF1* by BiFC in *N. Benthamiana*.
**Figure S4** Molecular characterization and phenotypes of *ZmPIF1* transgenic rice.
**Figure S5** Expression patterns of *ZmPIF3* in transgenic *Arabidopsis*.
**Figure S6** The germination rates of *ZmPIF1* transgenic rice and wild type seeds under NaCl and mannitol treatment.
**Figure S7** Hierarchical clustering analysis of all DEGs.
**Figure S8** Quantitative real‐time PCR validation of the results of DGE tag profiling.
**Figure S9** *ZmPIF1* transgenic rice showed wider tiller angle and more number of panicle phenotype.
**Table S1** Agronomic traits of *ZmPIF1* transgenic plants grown in the paddy field conditions in 2014.
**Table S2** Primer pairs used in quantitative real‐time PCR.
