# Supplementary material for: A maize phytochrome‐interacting factors protein ZmPIF1 enhances drought tolerance by inducing stomatal closure and improves grain yield in Oryza sativa
Source: Plant Biotechnol J. 2018 Mar 12;16(7):1375–87. doi: 10.1111/pbi.12878 (PMC5999191; doi:10.1111/pbi.12878)
Supplement: Supplementary file 1 — Figure S1 Sequence alignment of ZmPIF1 in other Zea mays and Arabidopsis PIFs family. Figure S2 Phylogenetic tree analysis of ZmPIF1 from other PIFs family. Figure S3 Confirmation of the subcellular localization of ZmPIF1 by BiFC in N. Benthamiana. Figure S4 Molecular characterization and phenotypes of ZmPIF1 transgenic rice. Figure S5 Expression patterns of ZmPIF3 in transgenic Arabidopsis. Figure S6 The germination rates of ZmPIF1 transgenic rice and wild type seeds under NaCl and mannitol treatment. Figure S7 Hierarchical clustering analysis of all DEGs. Figure S8 Quantitative real‐time PCR validation of the results of DGE tag profiling. Figure S9 ZmPIF1 transgenic rice showed wider tiller angle and more number of panicle phenotype. Table S1 Agronomic traits of ZmPIF1 transgenic plants grown in the paddy field conditions in 2014. Table S2 Primer pairs used in quantitative real‐time PCR. [file PBI-16-1375-s001.pdf]

A Maize phytochrome-interacting factors Protein ZmPIF1 Enhances Drought Tolerance by Inducing Stomatal Closure and Improves Grain Yield in Oryza sativa

Yong Gao, Meiqin Wu, Mengjiao Zhang, Wei Jiang, Xiaoyun Ren, Enxing Liang, Dongping Zhang, Changquan Zhang, Ning Xiao, Yan Li, Yi Dai, Jianmin Chen

Yong Gao and Meiqin Wu contributed equally to this work.

Corresponding author: Jianmin Chen

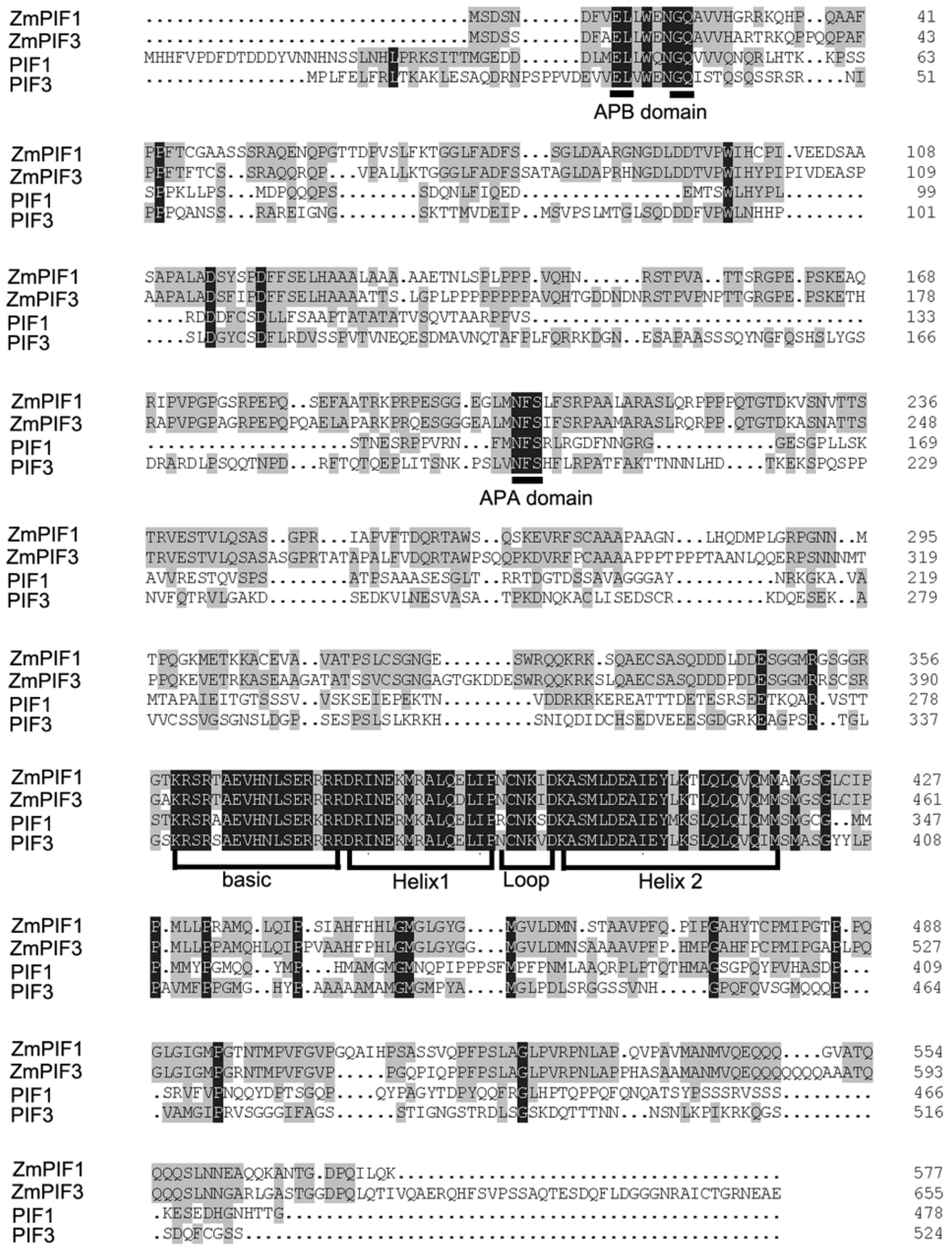

Figure S1 Sequence alignment of *ZmPIF1* in other *Zea mays* and *Arabidopsis* PIFs family. Comparison of the putative amino acid sequences of *ZmPIF1* with *Zea mays ZmPIF3* and *Arabidopsis thaliana* PIF1, PIF3 proteins. The conserved amino acids were showed in black with gray background. The specific sites for APB motif, APA motif and bHLH domain were presented. The aligned *ZmPIF3* (MaizeGDB accession no. GRMZM2G387528_T02) were from *Zea mays*, PIF1 (GenBank accession no. Q8GZM7.1), PIF3 (GenBank accession no. Q80536.1) sequences were from *Arabidopsis thaliana*.

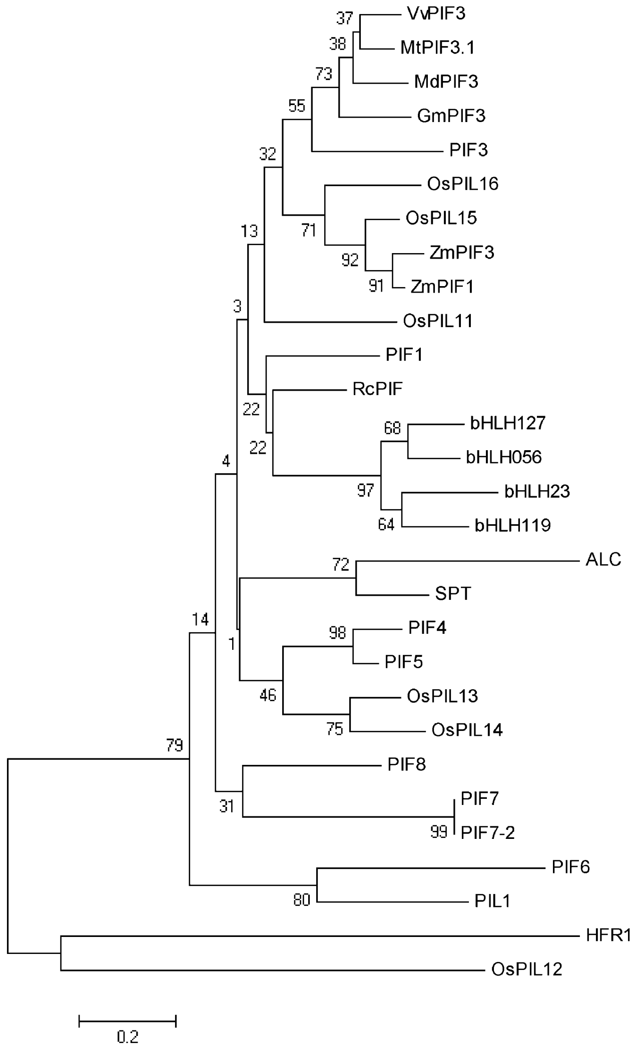

Figure S2 Phylogenetic tree analysis of *ZmPIF1* from other PIFs family. The phylogenetic tree was created in MEGA6 software with the neighbor-joining method based on the conserved domain of PIFs. Numbers indicate percentage Values after 1,000 replications. Sequences used in this analysis were as follows: *Arabidopsis thaliana* PIF1 (GenBank accession no. Q8GZM7.1); PIF3 (GenBank accession no. Q80536.1); PIF4 (GenBank accession no. Q8W2F3.1); PIF5 (GenBank accession no. Q84LH8.1); PIF6 (GenBank accession no. Q8L5W7.1); PIF7 (GenBank accession no. Q570R7.2); PIF7-2 (GenBank accession no. OAO94292); PIF8 (GenBank accession no. Q8GZ38.1); PIL1 (GenBank accession no. Q8L5W8.1); HFR1 (GenBank accession no. Q9FE22.1); bHLH127 (GenBank accession no. Q7XHI7.1); bHLH23 (GenBank accession no. Q9SVU6.1); bHLH119 (GenBank accession no. Q8GT73.2); bHLH056 (GenBank accession no. Q9SVU7.2); SPT (GenBank accession no. Q9FUA4.1); ALC (GenBank accession no. Q9FHA2.1); *Oryza sativa* OsPIL11 (GenBank accession no. NP_001067246); OsPIL12 (GenBank accession no. NP_001173558); OsPIL13 (GenBank accession no. NP_001051465); OsPIL14 (GenBank accession no. NP_001058876); OsPIL15 (GenBank accession no. NP_001042775); OsPIL16 (GenBank accession no. NP_001054593); *Vitis vinifera* VvPIF3 (XP_002276198); *Medicago truncatula* MtPIF3.1 (XP_003591254); *Malus domestica* MdPIF3 (GenBank accession no. AEX32796).; *Ricinus communis* RcPIF (XP_002521150); *Glycine max* GmPIF3 (GenBank accession no. XP_006589101); *Zea mays* ZmPIF3 (MaizeGDB accession no. GRMZM2G387528_T02).

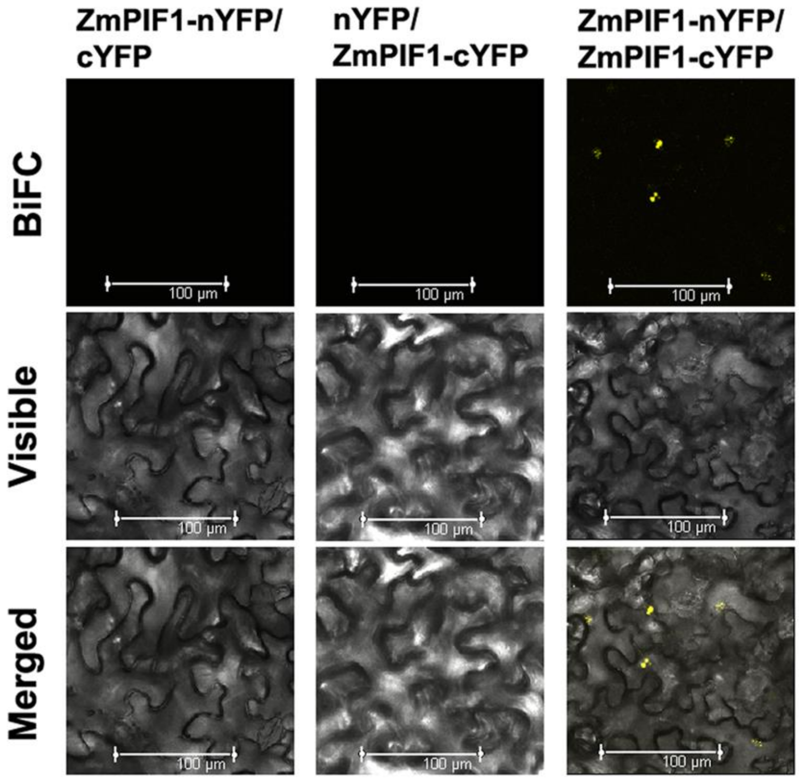

Figure S3 Confirmation of the Subcellular Localization of *ZmPIF1* by BiFC in *N. Benthamiana*. The transformed leaves were analyzed by confocal microscopy 48 h later. *ZmPIF1* was fused to the N-terminal region of yellow fluorescent protein and C-terminal region of yellow fluorescent protein, respectively. Bar=100 μm.

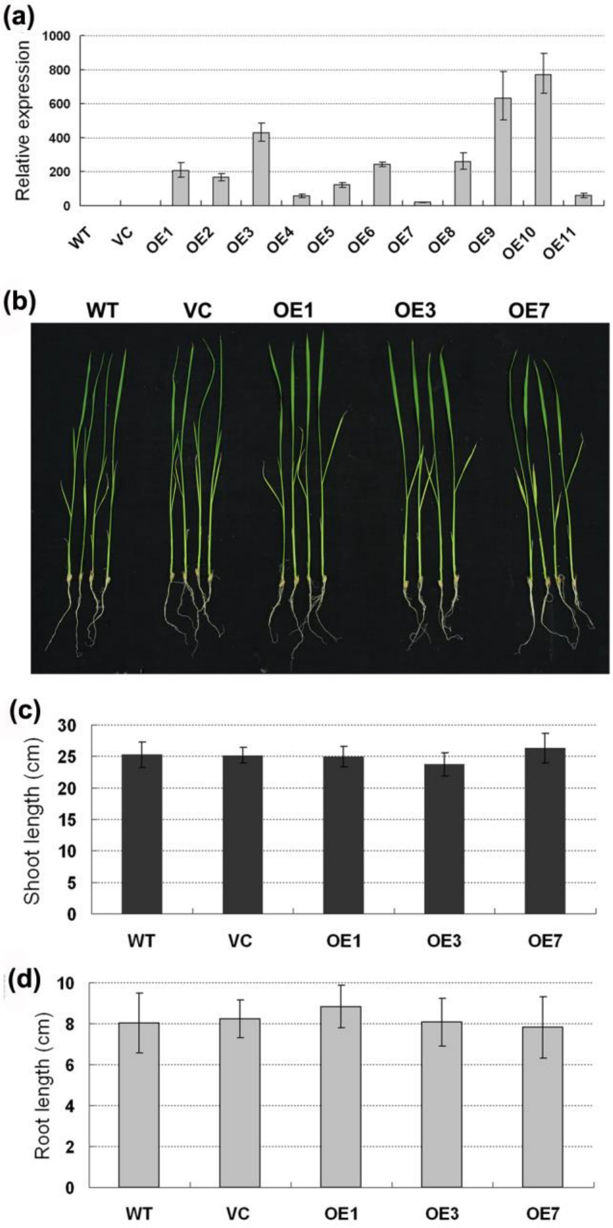

Figure S4 Molecular characterization and phenotypes of *ZmPIF1* transgenic rice. (a) *ZmPIF1* expression in wild-type, vector control and transgenic rice. Total RNAs from two-week-old wild type, vector control and transgenic plants were isolated, reverse-transcribed, and analyzed by quantitative real-time PCR (qRT-PCR). Actin was used as an internal control. Error bars are based on three replicates. (b) The phenotypes of the T3 generation of wild-type and transgenic plants after growing on 1/2 MS medium for 14 days. (c, d) Comparison of shoot and root lengths for *ZmPIF1* transgenic rice. Ten seeds of each line were planted in triplicate. Actual measurements of shoot and root length are compared in (c) and (d).

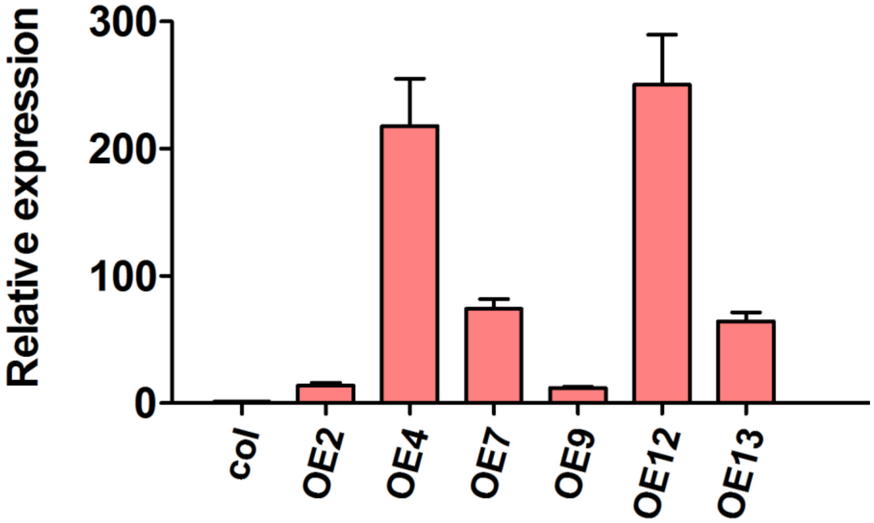

Figure S5 Expression patterns of *ZmPIF3* in transgenic *Arabidopsis*. col: *Arabidopsis thaliana* L. Heynh, Columbia; OE2, OE4, OE7, OE9, OE12, OE13, *ZmPIF1* transgenic lines. Total RNAs from col and transgenic *Arabidopsis* were isolated, reverse-transcribed, and analyzed by quantitative real-time PCR (qRT-PCR). Actin was used as an internal control. Error bars are based on three replicates.

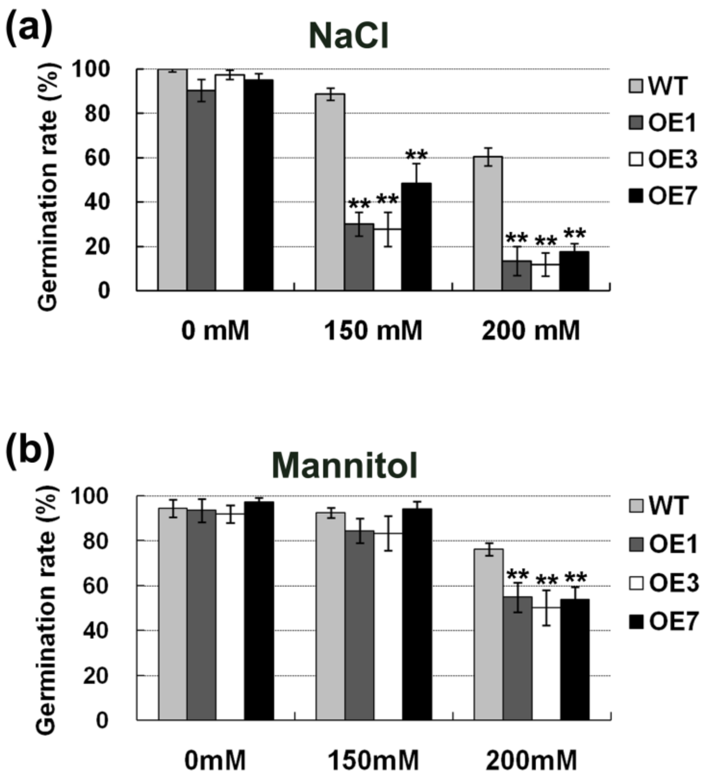

Figure S6 The germination rates of *ZmPIF1* transgenic rice and wild type seeds under NaCl and mannitol treatment. (a) The germination rates of ZmPIF1 transgenic rice and wild type seeds on wet filter paper containing 0, 150, 200mM NaCl for 7 d (n=35). (b) The germination rates of ZmPIF1 transgenic rice and wild type seeds on wet filter paper containing 0, 150, 200mM mannitol for 7d (n=35).

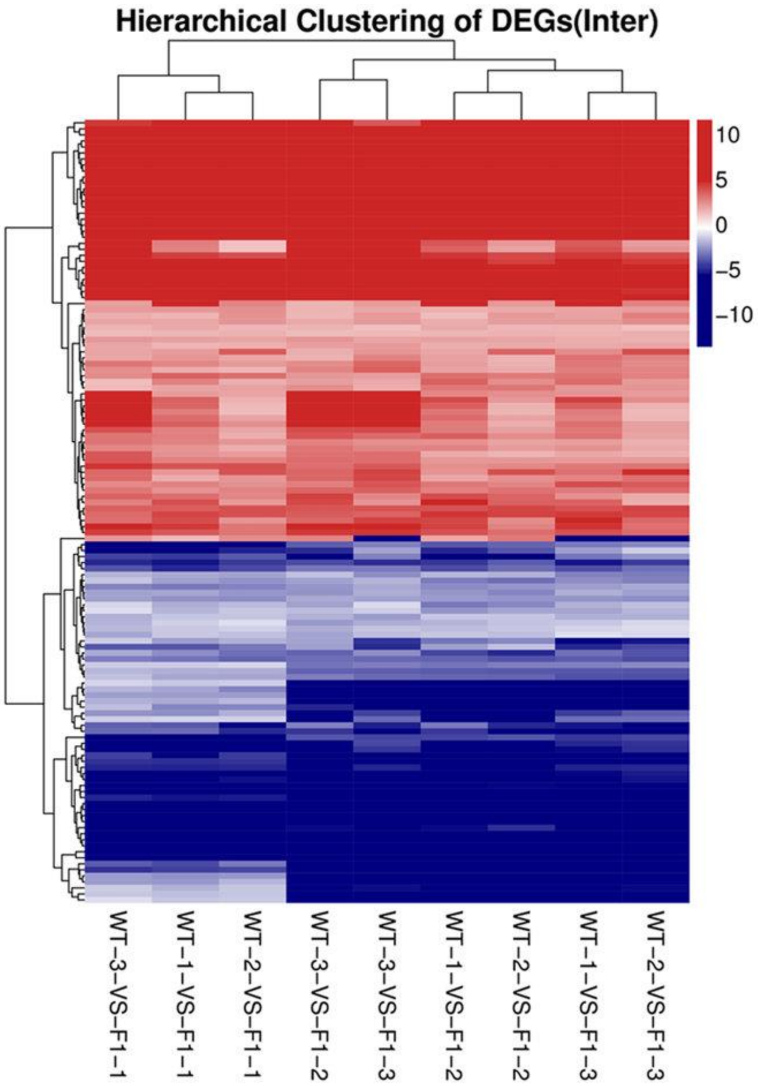

Figure S7 Hierarchical clustering analysis under common conditions of expression patterns of genes that were differentially expressed in three independent *ZmPIF1* transgenic rice lines compared with wild-type rice based on the expression data. Red and blue colors indicate up-regulation and down-regulation of the genes, respectively, in *ZmPIF1* transgenic rice. WT-1, WT-2 and WT-3, wild type; F1-1, F1-2 and F1-3, three *ZmPIF1* transgenic lines.

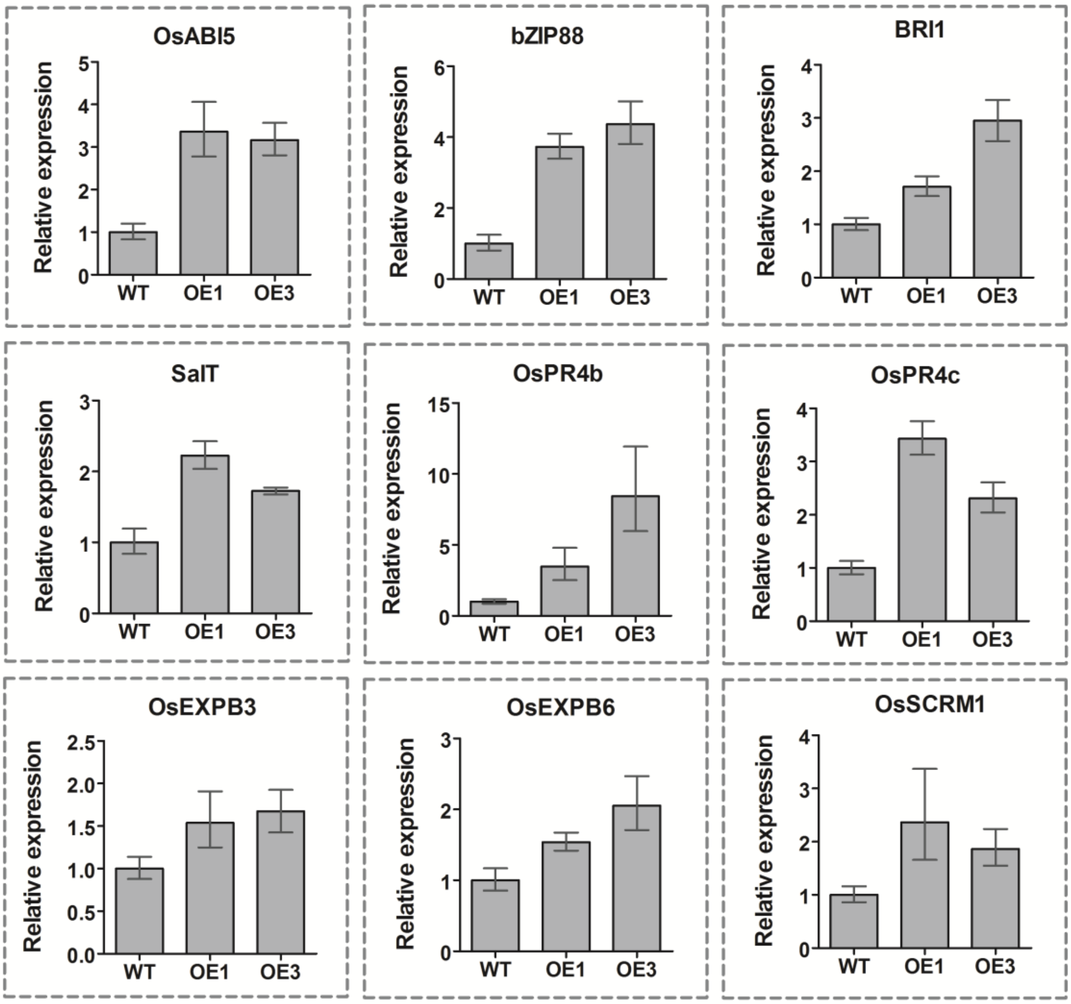

Figure S8 Quantitative real-time PCR validation of the results of DGE tag profiling. Ten seedlings were pooled as a sample.

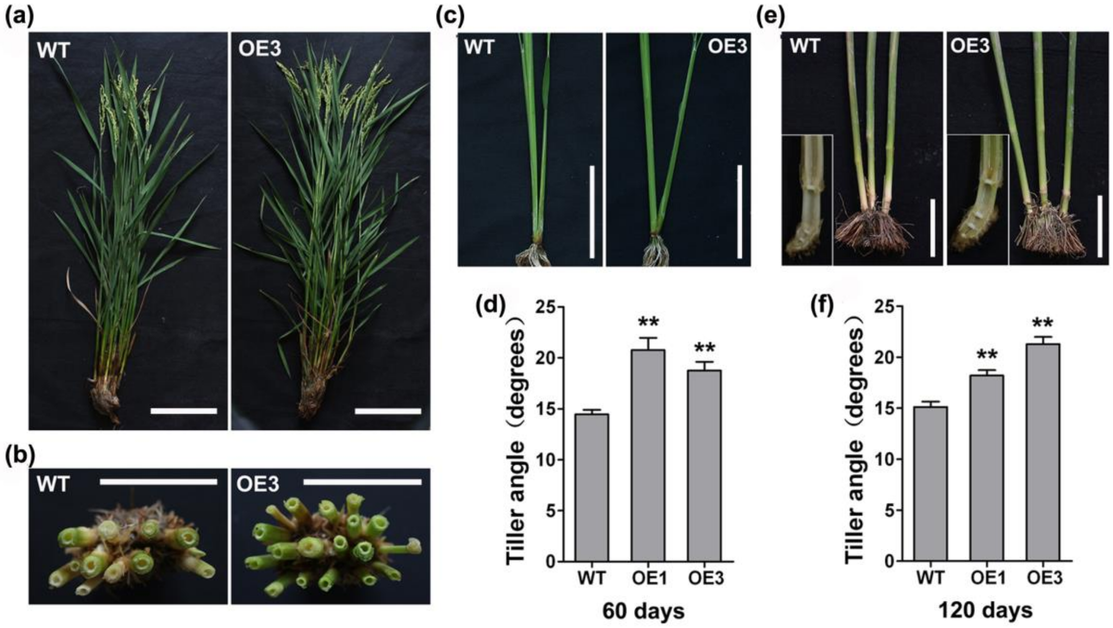

Figure S9 *ZmPIF1* transgenic rice showed wider tiller angle and more number of panicle phenotype. (a) Phenotype of *ZmPIF1* transgenic rice and wild type plants at 120 days. Bar=20cm. (b) Phenotype of the tiller base of *ZmPIF1* transgenic rice and wild type plants at 120 days. Bar=3cm. (c) Phenotype of the angle between main culm and the first side tiller of *ZmPIF1* transgenic plants and wild type plants at 60 days. Bar=10cm. (d) Tiller angle of *ZmPIF1* transgenic rice and wild type plants at 60 days. (e) Phenotype of the tiller base (left panels) and angle between main culm and the first side tiller of *ZmPIF1* transgenic rice and wild type plants at 120 days. Bar=10cm. (f) Tiller angle of *ZmPIF1* transgenic rice and wild type plants at 120 days. (d, f) Data represent the mean ± SE. ** t test, with $P < 0.01$; * t test, with $P < 0.05$.

Table S1 Agronomic traits of *ZmPIF1* transgenic plants grown in the paddy field conditions in 2014.

Lines	Panicle Number per Plant	Panicle Length (cm)	No. of Grains per Panicle	Filled Grains per Panicle	Seed-Setting Rate (%)	1,000-Grain Weight (g)
**2014**						
**WT**	8.3±2.08	16.5±0.80	143.9±24.5	128.5±24.2	89.2±3.94	26.86±0.12
**VC**	9.7±2.52	16.3±0.27	152.9±13.8	136.8±12.3	89.5±2.81	26.91±0.11
ZmPIF1						
**OE1**	10.5±4.73	16.1±0.78	138.1±10.9	127.6±11.4	92.5±5.55	26.62±0.01
**OE3**	12.3±2.08	16.4±0.76	158.8±16.0	136.4±12.7	86.0±2.14	25.45±018**
**OE7**	14.8±3.61*	16.0±0.64	144.9±30.5	124.3±25.3	86.0±5.12	25.43±0.22**

WT, wild type; VC, vector control; OE1, OE3 and OE7, three *ZmPIF1* transgenic plants. Values are mean ± SD (n>3). * and ** indicate significant differences at P <0.05 and P <0.01, respectively.

Table S2 Primer sequences used in this study.

Primer name	Forward primer	Reverse primer
**ZmPIF1 Clone**	5’-GAAGATCTATGTCCGACAGCAACGACT-3’	5'-CTATTTTTGTAGTATTTGTGGATCTC-3'
**ZmPIF1 qRT-PCR**	5’-GCAGTCGCTACTCCATCGC-3’	5’-TCCTCGCATCCCACCAGAC-3’
**Actin**	5'- GCATCCATGAGACCACCTACAAC -3'	5'- GATGGACCCTCCTATCCAGACAC -3'
**ZmPIF1 Subcellular localization**	5’-AATAAAGCTTATGTCCGACAGCAACGAC-3’	5’-AATACTCGAGTTTTTGTAGTATTTGTGGATCTC-3
**ZmPIF1 BiFC**	5’-GGGGTACCATGTCCGACAGCAACGACT-3’	5'-GCTCTAGACTATTTTTGTAGTATTTGTGGATCTC-3'
**ZmPIF1 overexpression vector construction in rice**	5’-GAAGATCTATGTCCGACAGCAACGACT-3’	5’-CGGGGTACCCTATTTTTGTAGTATTTGTGGATCTC-3’
**ZmPIF1 overexpression vector construction in *Arabidopsis***	5’-GGGGTACCATGTCCGACAGCAACGACT-3’	5’-GAAGATCTCTATTTTTGTAGTATTTGTGGATCTC-3’
**bZIP88 qRT-PCR**	5’-ACTGGACAAACTGTAGCCTCCC-3’	5'-GCTGATGCTGCCTCAAAATG-3'
**OsABI5 qRT-PCR**	5’-AGTGGCACACTCATCCTGCTT-3’	5'-TGAAAGAAGACTTAATGTGCAAATCC-3'
**BRI qRT-PCR**	5’-TGGCAAGGTTTACAAGGGTC-3’	5'-GATGCTGAACCATGCGTAGA-3'
**SalT qRT-PCR**	5’-TGGATTCTTTGGAAGGTCTGG-3’	5'-TTGACCACTGGGAATCAAGG-3'
**OsPR4b qRT-PCR**	5’-TATGGCCATGGCACAAGAGG-3’	5'-CGTGATCTGATCCCCTGTCG-3'
**OsPR4c qRT-PCR**	5’-TCGTGGCGTCAGAAGTATGG-3	5'-ACGGTGTCCCAGTCCAGG-3'
**OsEXPB3 qRT-PCR**	5’-GCACTCTTCTCCTTCCTTGTTG-3	5'-CCTTGAAGATAGGCTCGTTGC-3'
**OsEXPB6 qRT-PCR**	5’-CAGCCAGAATGGGCAGCA-3	5'-CGCAGGAGGTCATGGACGA-3
**OsSCRM1 qRT-PCR**	5’-GCTCAACGACCGCCTCTACA-3	5'-GGTGTTGGAGGCAATGACG-3
